# Supplementary material for: Synthesis and In Vitro Photocytotoxicity of 9-/13-Lipophilic Substituted Berberine Derivatives as Potential Anticancer Agents
Source: Molecules. 2020 Feb 5;25(3):677. doi: 10.3390/molecules25030677 (PMC7036939; doi:10.3390/molecules25030677)
Supplement: Supplementary file 1 [file molecules-25-00677-s001.pdf]

molecules

MDPI

Supporting Information

Synthesis and in vitro photocytotoxicity of 9-/13-lipophilic substituted berberine derivatives as potential anticancer agents

1. General methods

All chemical reagents in commercial quality were used as received (Sigma-Aldrich, St. Louis, MO, USA), Fluka (Roma, Italy), TCI (Tokyo, Japan), and Alfa Aesar without further purification. Solvents were dried and the synthesized compounds were purified using standard techniques. Reactions-progression was monitored by TLC on aluminum plates coated with silica gel with a fluorescent indicator (Merck 60 F_{254}). Unless otherwise stated. Melting points were determined on in open capillaries using the Fargo MP-2D apparatus and are uncorrected. NMR spectra were recorded using TMS as an internal standard in $CDCl_3$ at 500.13 MHz for ^{1}H and at 125.77 MHz for ^{13}C (Bruker Biospin GmbH AVANCE III 500 MHz, Rheinstetten, Germany). Chemical shift (δ) were reported in parts per million (ppm) measured relative to the internal standards (TMS), and the coupling constant (J) were expressed in Hertz (Hz). Column chromatography was performed with silica gel Silia*Flash*® G60 (60–200 μm) purchased from SiliCycle Inc. (Quebec City, QC, Canada). In general, the reactions were carried out under anhydrous conditions in dry solvent and nitrogen atmosphere. The purity of these compounds was based on the analysis of HPLC (Hitachi High-Technologies, Tokyo, Japan) equipped with a 280 nm detector and LiChroCART RP-C_{18} column (4.6 mm i.d. × 250 mm, 5 μm, Merck, Darmstadt, Germany). The mobile phase was composed of MeOH-H_2O (0.05% TFA) (90:10) and the flow rate was 1.0 mL/min. The purity of all compounds was more than 98%. The mass spectra were acquired using a Thermo Finnigan model LXQ (Thermo Electron Co., Waltham, MA, USA) ion trap mass spectrometer equipped with ESI source interference and controlled by Xcalibur 2.06. The mass spectra were acquired in a positive ion mode or a negative ion mode.

2. Extraction and isolation

Take 150 g of *Phellodendron amurense* Ruprecht and percolate with 500 mL of methanol for 3 h, with another 300 mL repeat the process twice. Combine the extracts and evaporate the solvent to 100 mL. To the concentrated extract add 1300 mL of water with stirring. Remove the resinous substance by filtration, then concentrated the filtrate to 300 mL and react with 60 mL of 10% hydrochloric acid. Let the solution stand for several hours and crude crystals of hydrochloride salt are obtained. Recrystallize with 95% ethanol and approximately 2.5 g of yellow needle-like crystals of berberine chloride **1** are obtained, yellow solid; UV (MeOH) λ_{max} (log ε) 429 (3.85), 349 (4.50), 266 (4.53), 230 (4.54), 202 (4.39) nm; ^{1}H NMR (500 MHz, DMSO-d_6) δ: 9.89 (s, 1H, H-8), 8.94 (s, 1H, H-13), 8.20 (d, J = 9.1 Hz, H-12), 7.99 (d, J = 9.1 Hz, 1H, H-11), 7.79 (s, 1H, H-1), 7.08 (s, 1H, H-4), 6.17 (s, 2H, -OCH_2O-), 4.93 (t, J = 6.1 Hz, 2H, H-6), 4.10 (s, 3H, OCH_3), 4.07 (s, 3H, OCH_3), 3.20 (t, J = 6.1 Hz, 2H, H-5); ^{13}C NMR (125 MHz, DMSO-d_6) δ: 150.4, 149.8, 147.7, 145.4, 143.7, 137.5, 133.0, 126.8, 123.5, 121.4, 120.4, 120.2, 108.4, 105.4, 102.1, 61.9, 57.1, 55.2, 26.3; LC-MS (ESI$^+$, m/z) $C_{20}H_{18}NO_4^+$: 336.22 $[M-Cl]^+$.

3. Chemical synthesis of berberrubine (2)

The pyrolysis of berberine **1** (5.0 g) was heated at 190°C under vacuum (20–30 mmHg) for 30–60 min. The reaction mixture was chromatographed on a silica gel column and eluted with ethyl acetate/methanol (2/1) solvent to yield 3.8 g of berberrubine **2** as a red powder, yield: 79%; UV (MeOH) λ_{max} (log ε) 511 (3.68), 390 (4.03), 329 (3.98), 278 (4.34), 239 (4.43), 213 (4.24) nm; ^{1}H NMR (500 MHz, DMSO-d_6) δ: 9.06 (s, 1H, H-8), 7.97 (s, 1H, H-13), 7.60 (s, 1H, H-1), 7.21 (d, J = 7.9 Hz, H-12), 6.95 (s, 1H, H-4), 6.35 (d, J = 7.9 Hz, 1H, H-11), 6.09 (s, 2H, -OCH_2-), 4.47 (t, J = 6.1 Hz, 2H, H-6), 3.72 (s, 3H,

-OCH3), 3.03 (t, *J* = 6.1 Hz, 2H, H-5); ^{13}C NMR (125 MHz, DMSO-*d*6) δ: 167.3, 149.7, 148.3, 147.3, 145.8, 133.2, 132.0, 129.2, 121.8, 121.3, 120.0, 117.0, 108.3, 104.7, 101.6, 100.7, 55.7, 52.3, 27.5; LC-MS (ESI+, *m*/*z*) $C_{19}H_{16}NO_4^+$: 322.29 [M – Cl]+.

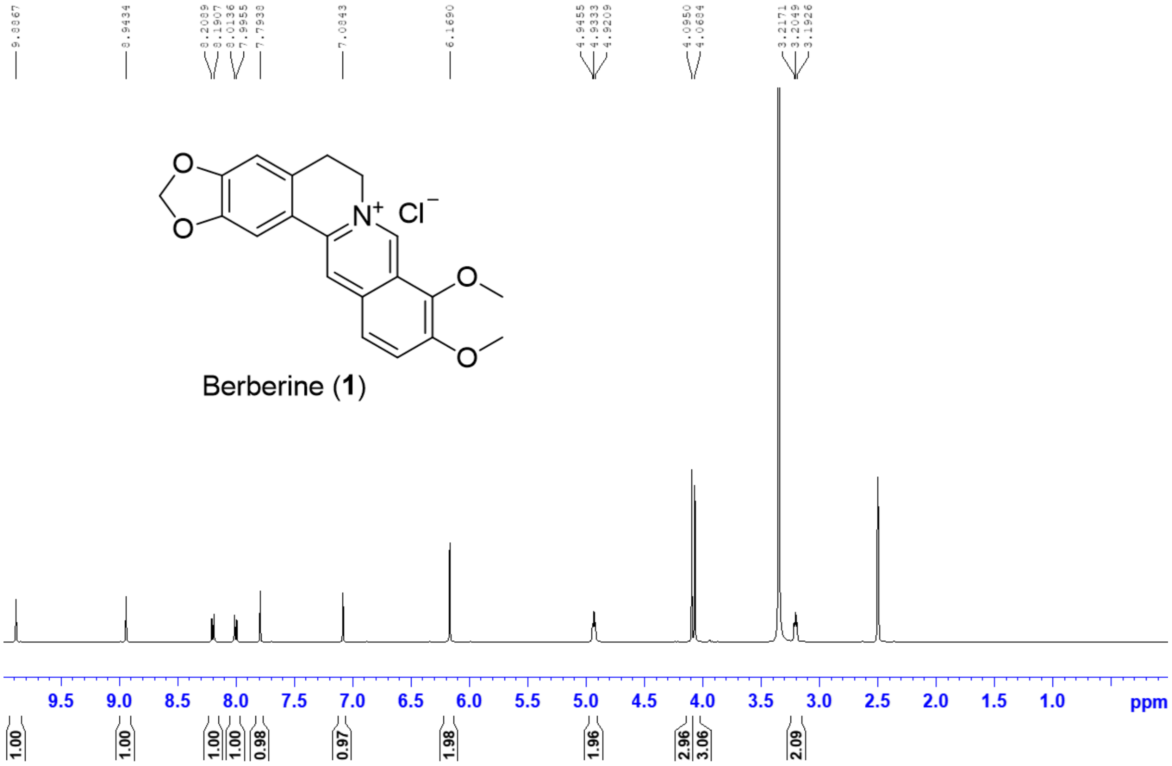

(**A**)

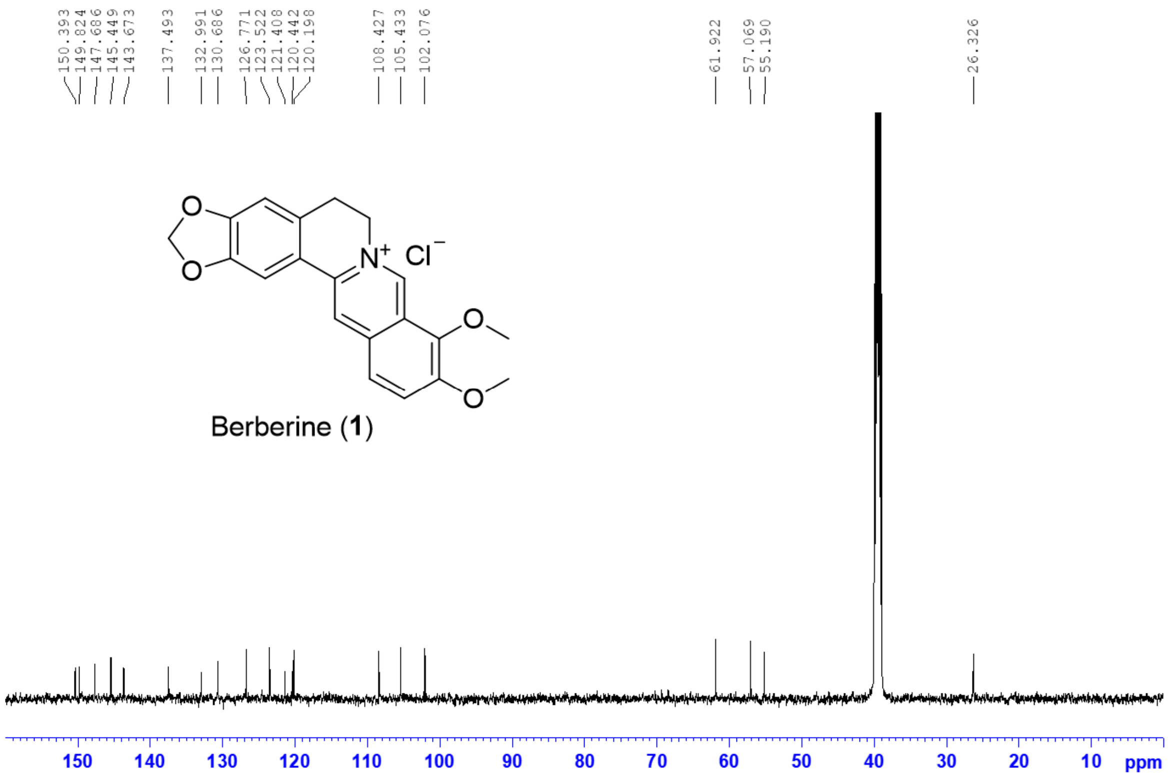

(**B**)

Figure S1. (A) ^{1}H NMR spectra of berberine (**1**); (B) ^{13}C NMR spectra of berberine (**1**).

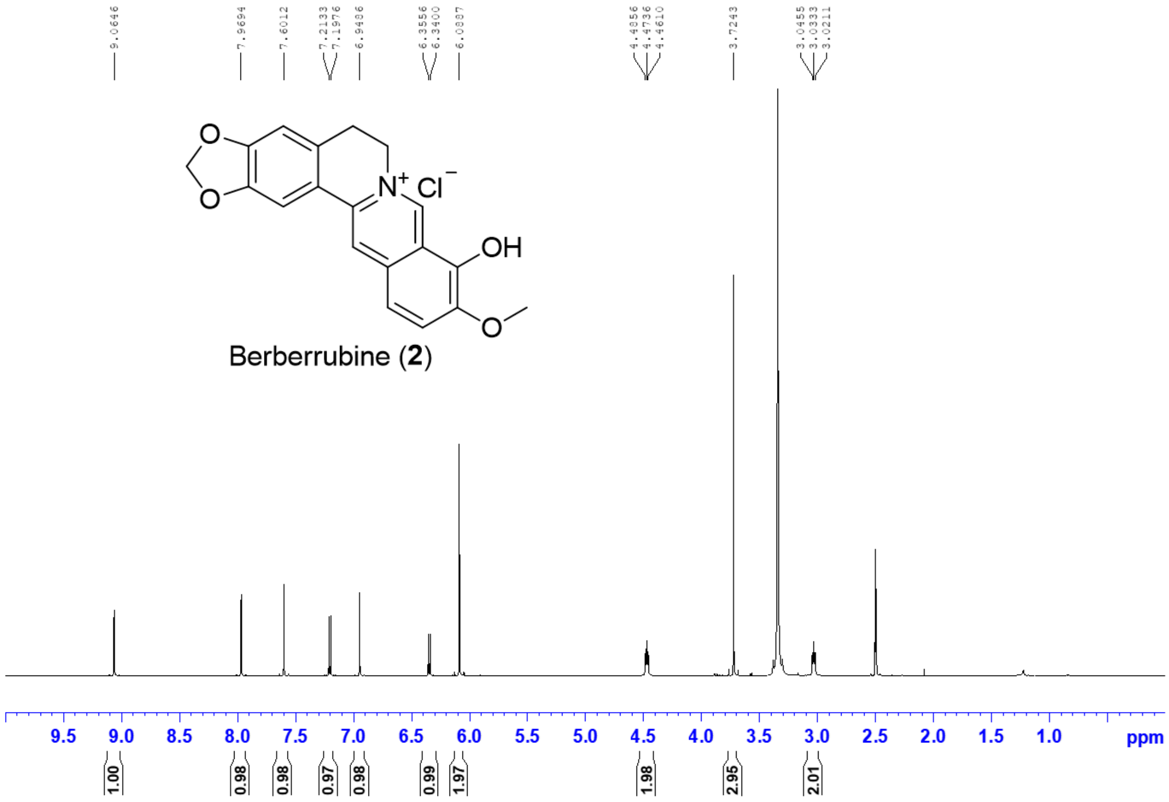

(**A**)

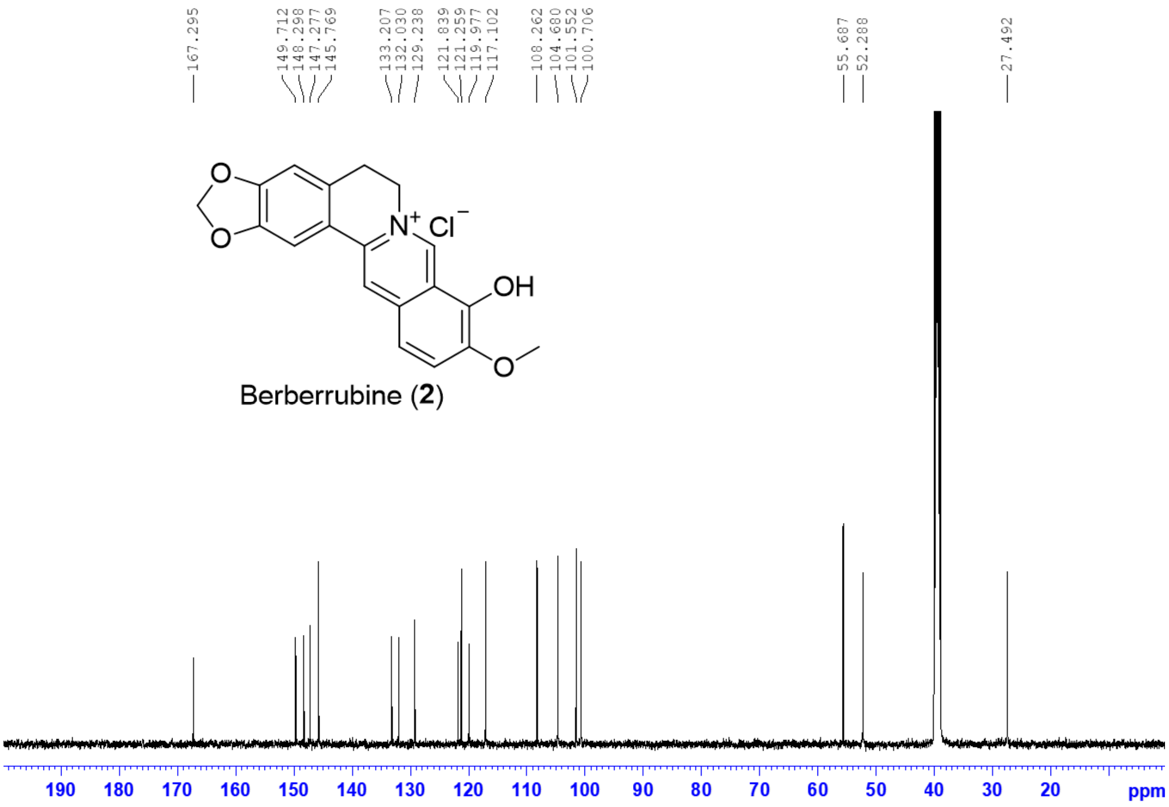

(**B**)

Figure S2. (A) ^{1}H NMR spectra of berberrubine (**2**); (B) ^{13}C NMR spectra of berberrubine (**2**).

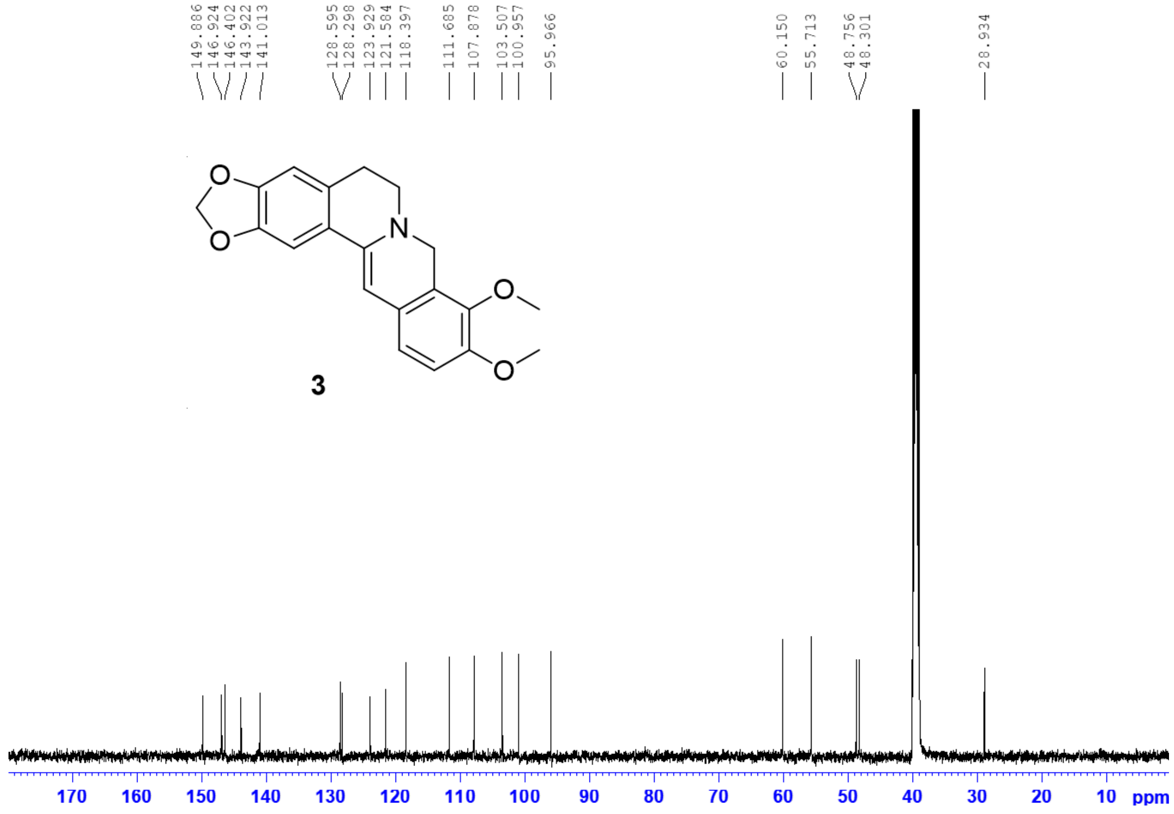

(**A**)

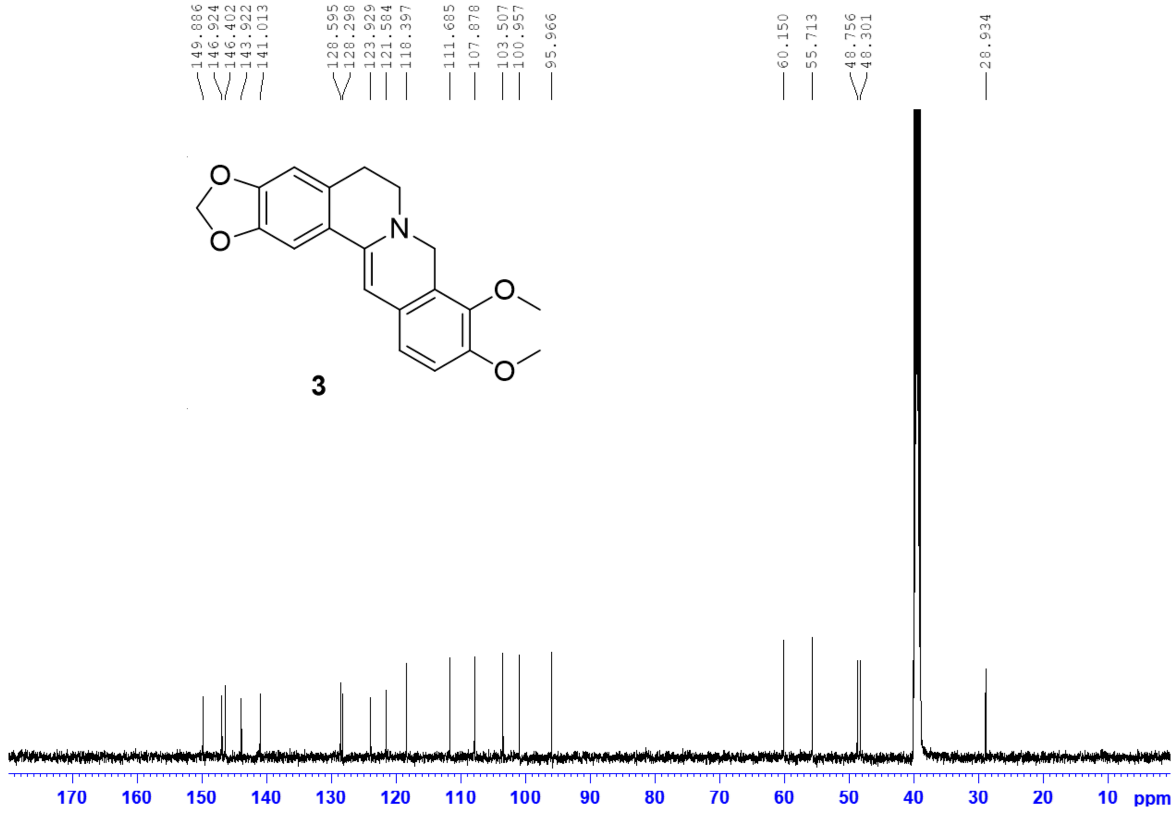

(**B**)

Figure S3. (A) ^{1}H NMR spectra of 8-dihydroberberine (**3**); (B) ^{13}C NMR spectra of 8-dihydroberberine (**3**).

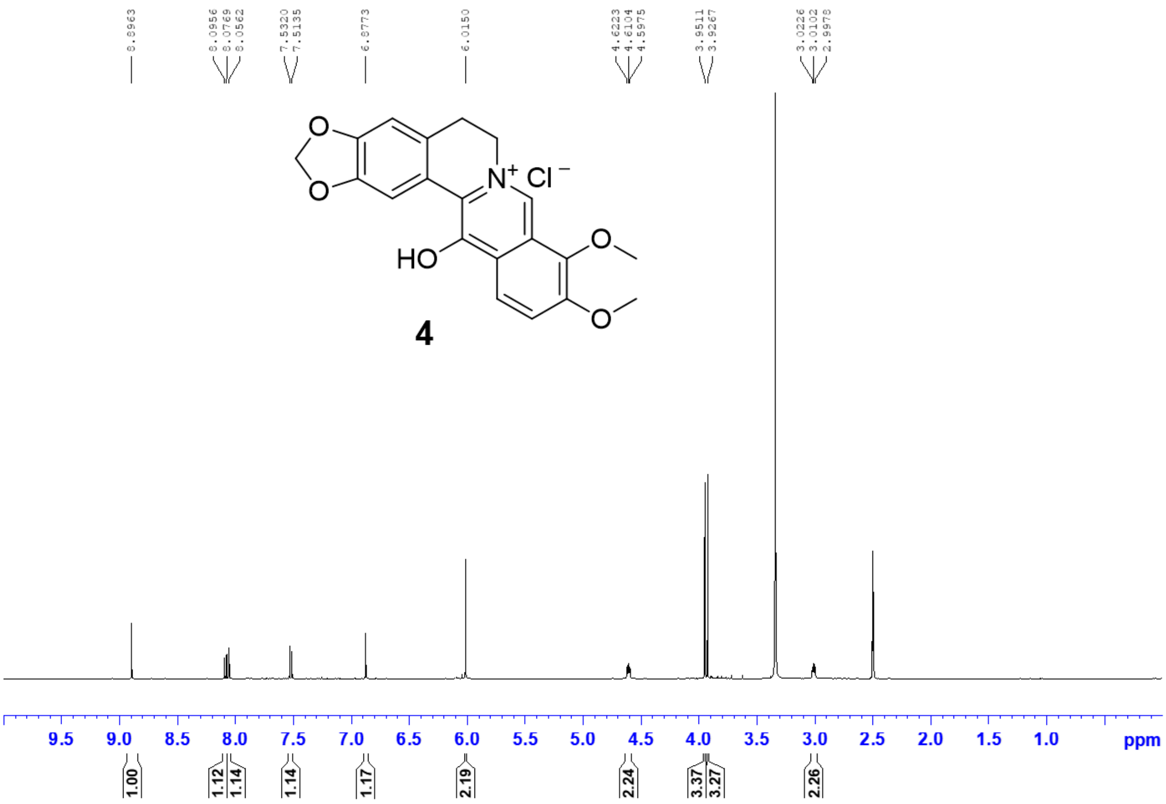

(**A**)

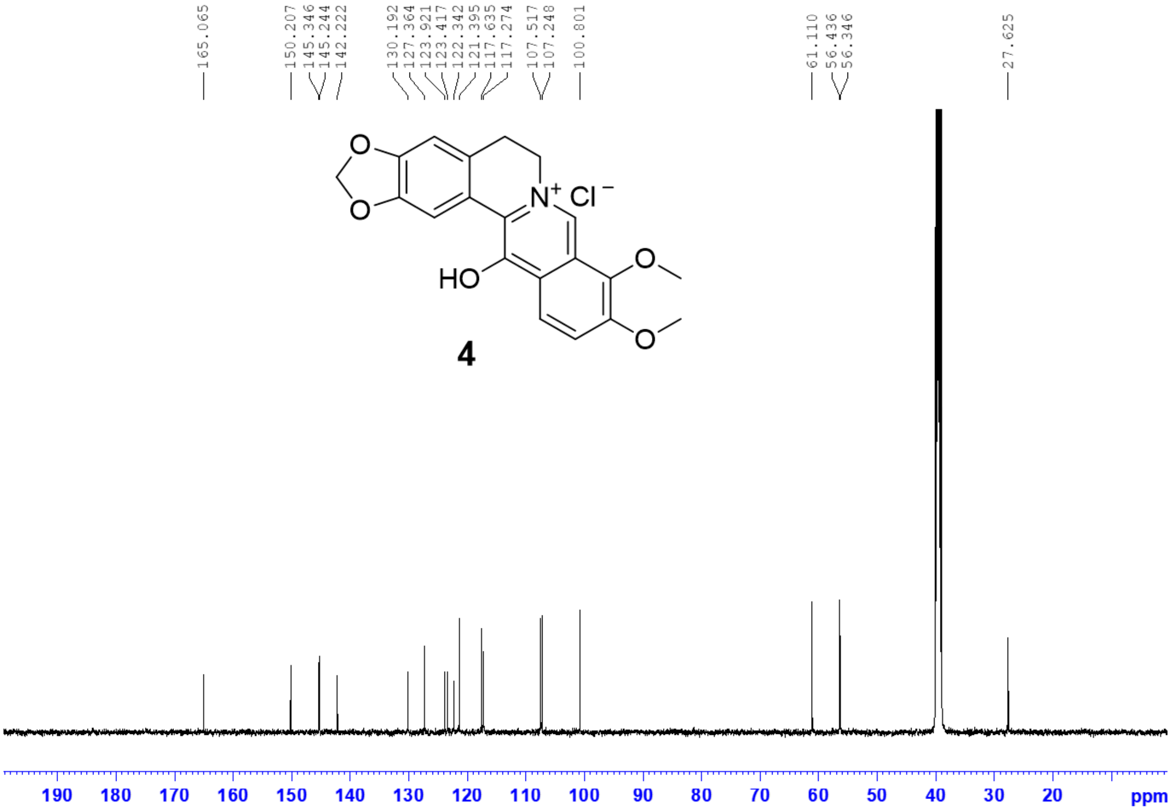

(**B**)

Figure S4. (A) ^{1}H NMR spectra of 13-hydroxyberberine (**4**); (B) ^{13}C NMR spectra of 13-hydroxyberberine (**4**).

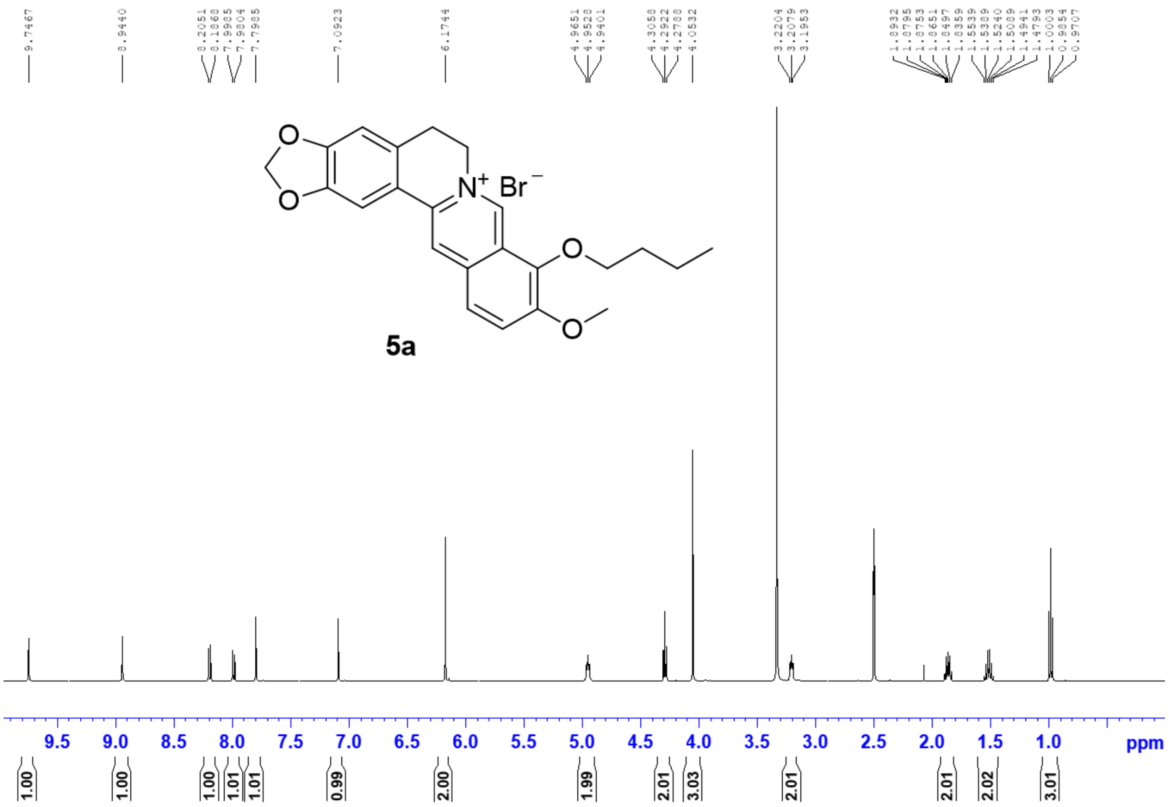

(**A**)

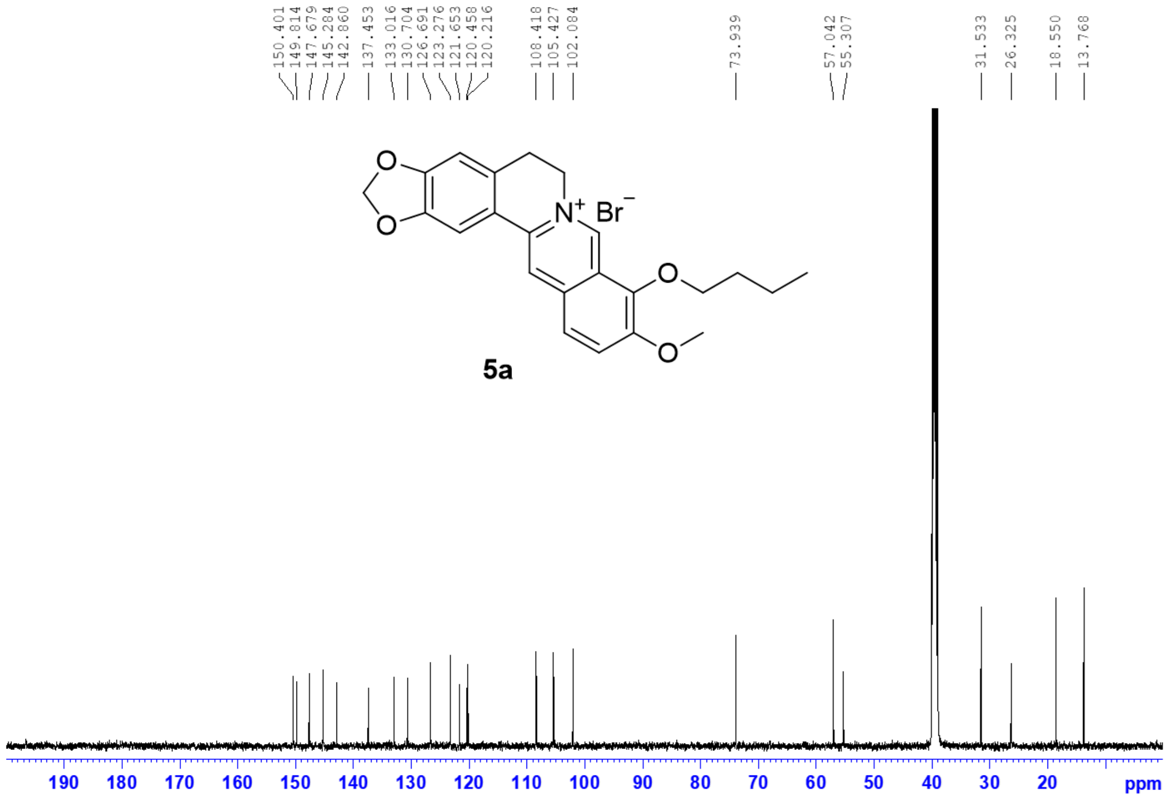

(**B**)

Figure S5. (A) ^{1}H NMR spectra of 9-*O*-butylberberine (**5a**); (B) ^{13}C NMR spectra of 9-*O*-butylberberine (**5a**).

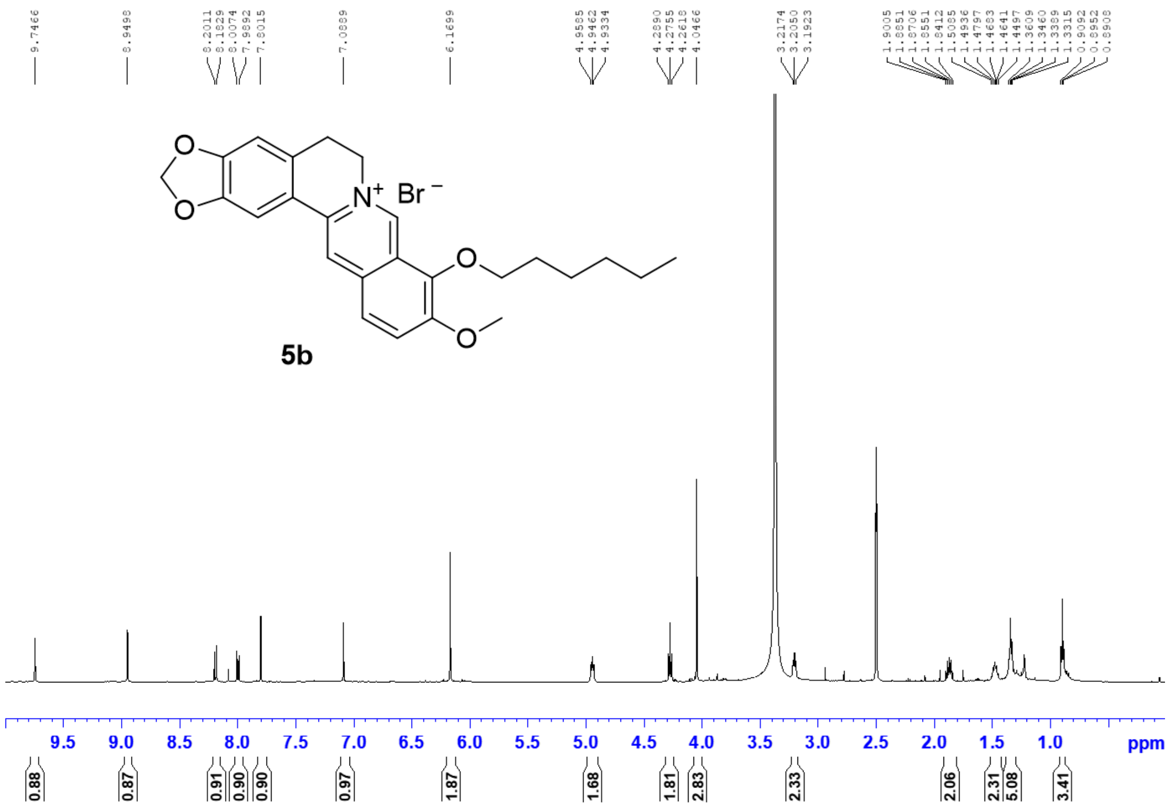

(**A**)

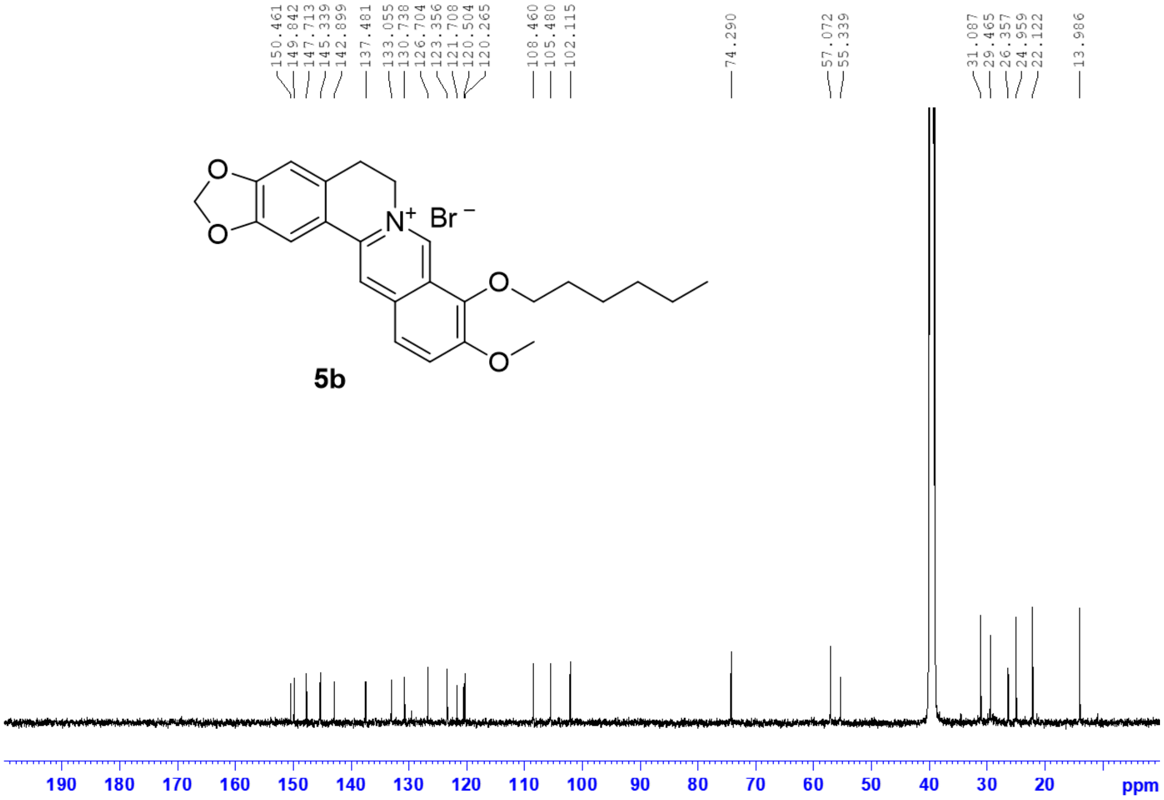

(**B**)

Figure S6. (A) ^{1}H NMR spectra of 9-*O*-hexylberberine (**5b**); (B) ^{13}C NMR spectra of 9-*O*-hexylberberine (**5b**).

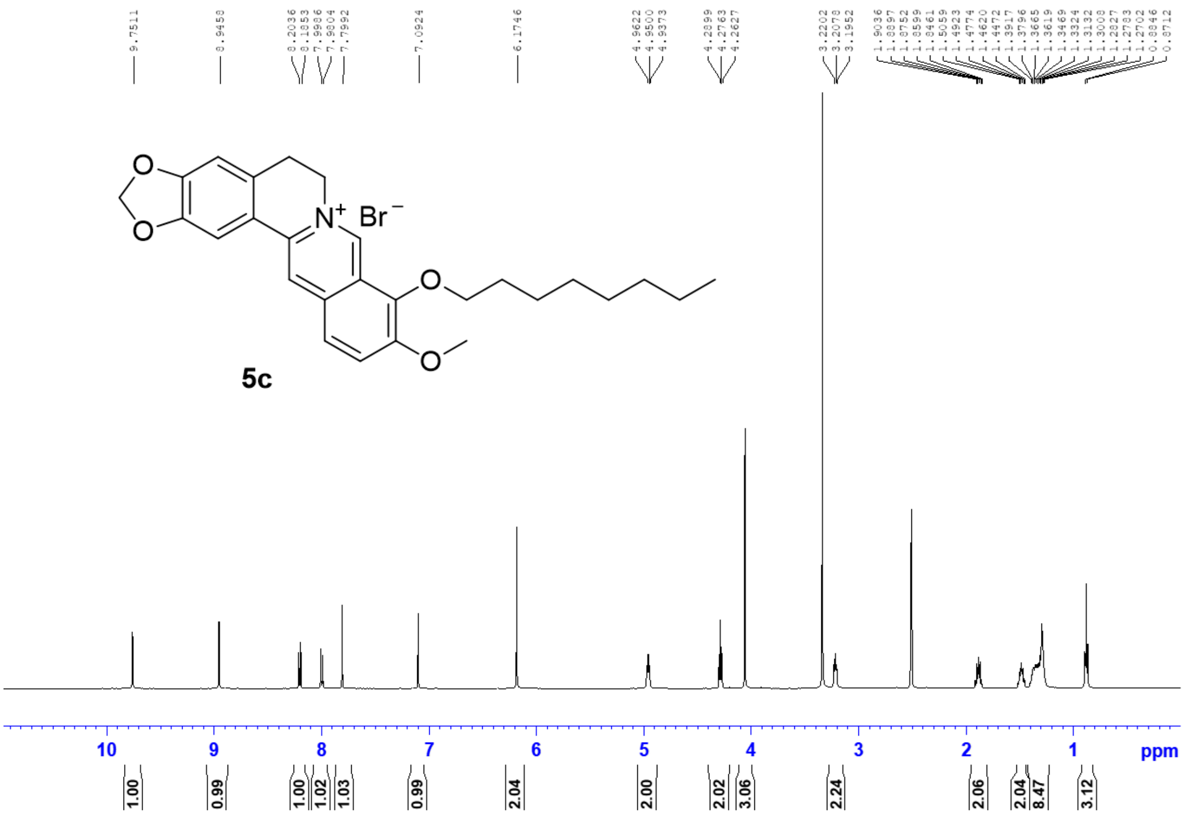

(**A**)

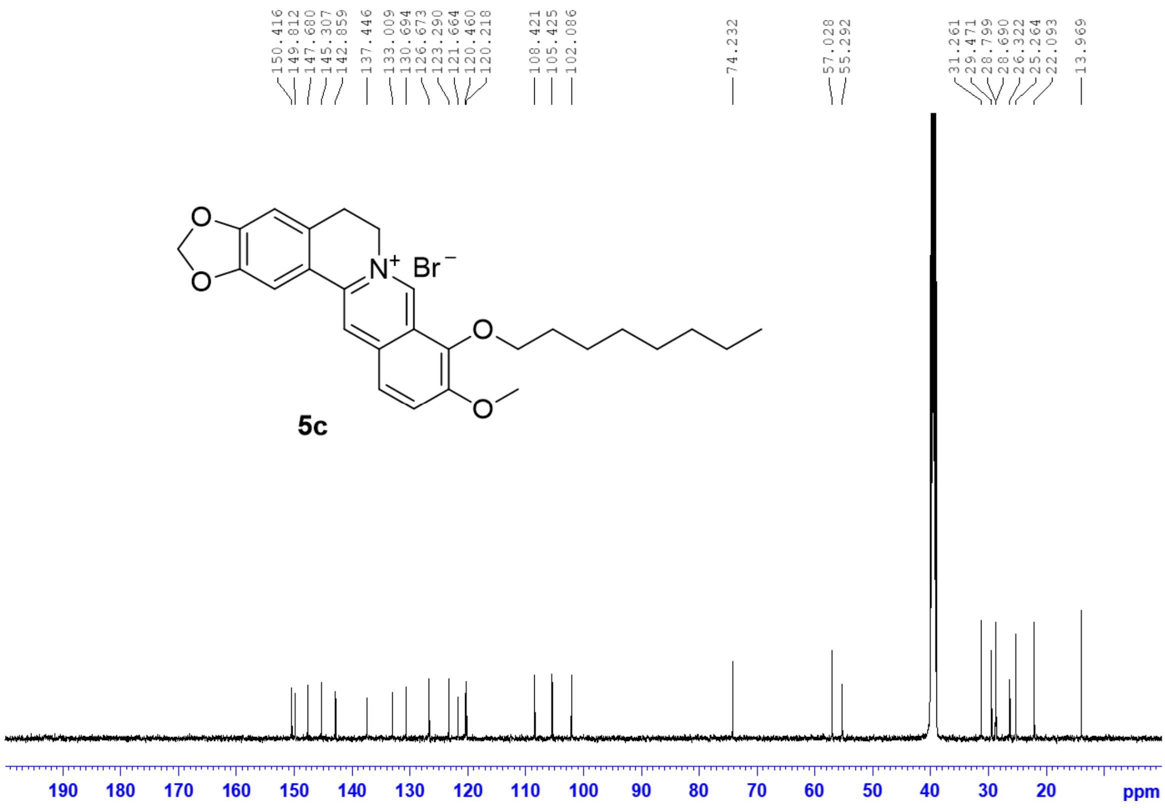

(**B**)

Figure S7. (A) ^{1}H NMR spectra of 9-*O*-octylberberine (**5c**); (B) ^{13}C NMR spectra of 9-*O*-octylberberine (**5c**).

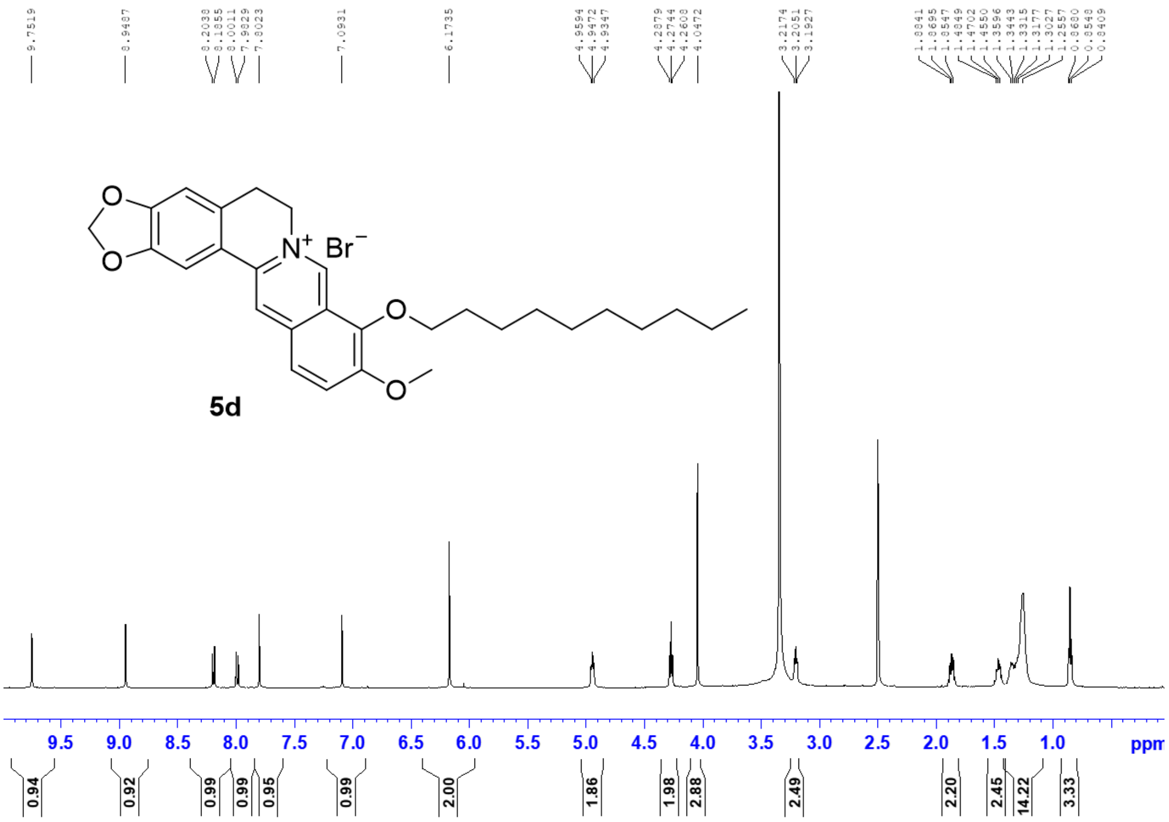

(**A**)

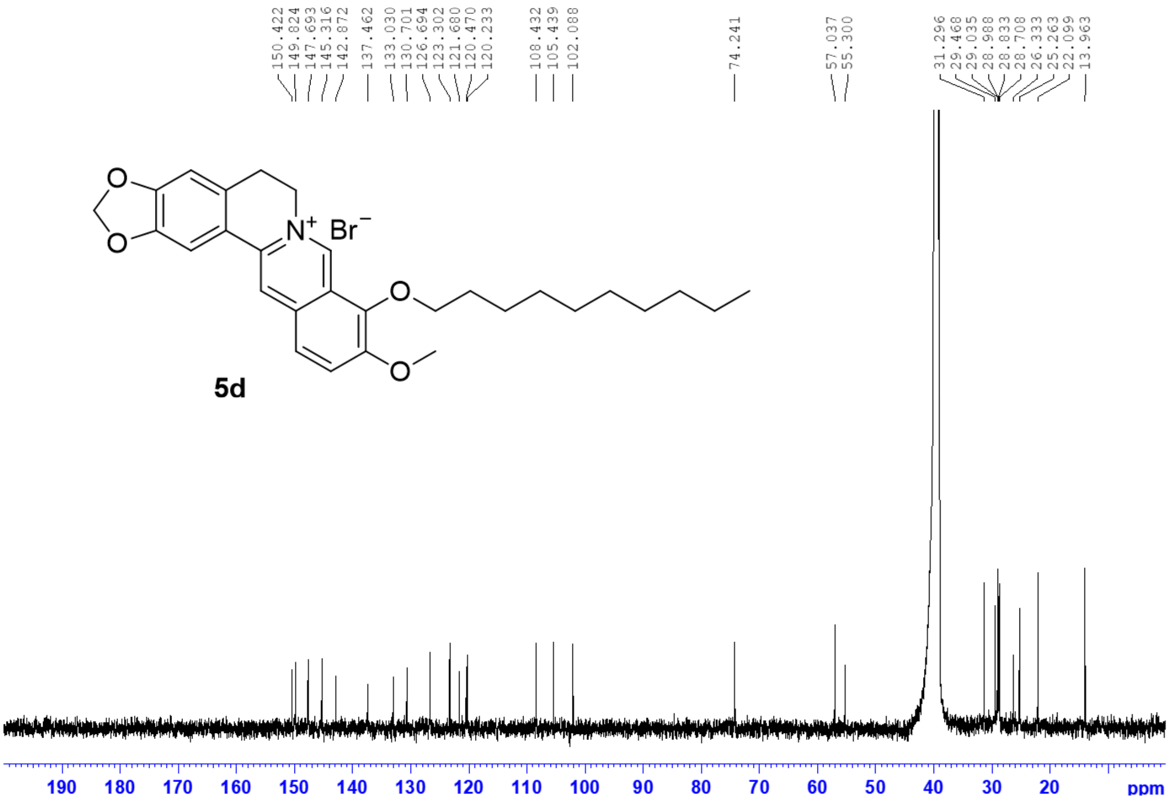

(**B**)

Figure S8. (A) ^{1}H NMR spectra of 9-*O*-decylberberine (**5d**); (B) ^{13}C NMR spectra of 9-*O*-decylberberine (**5d**).

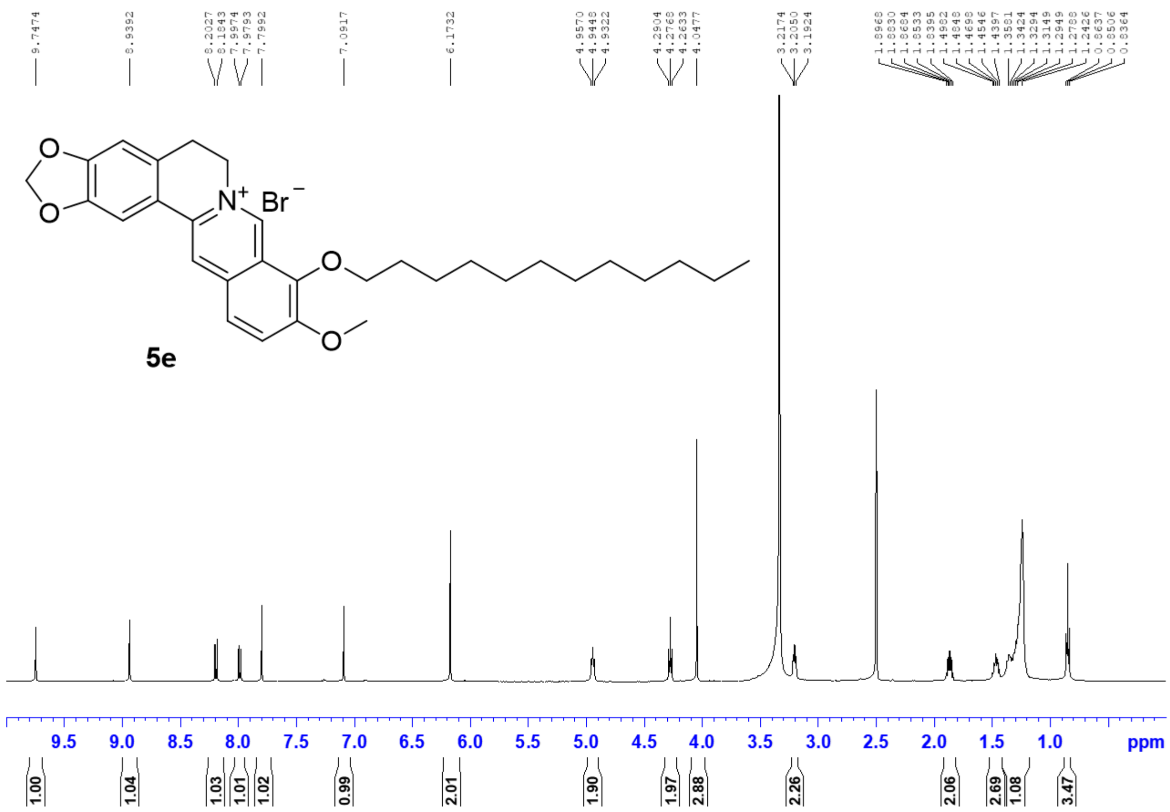

(**A**)

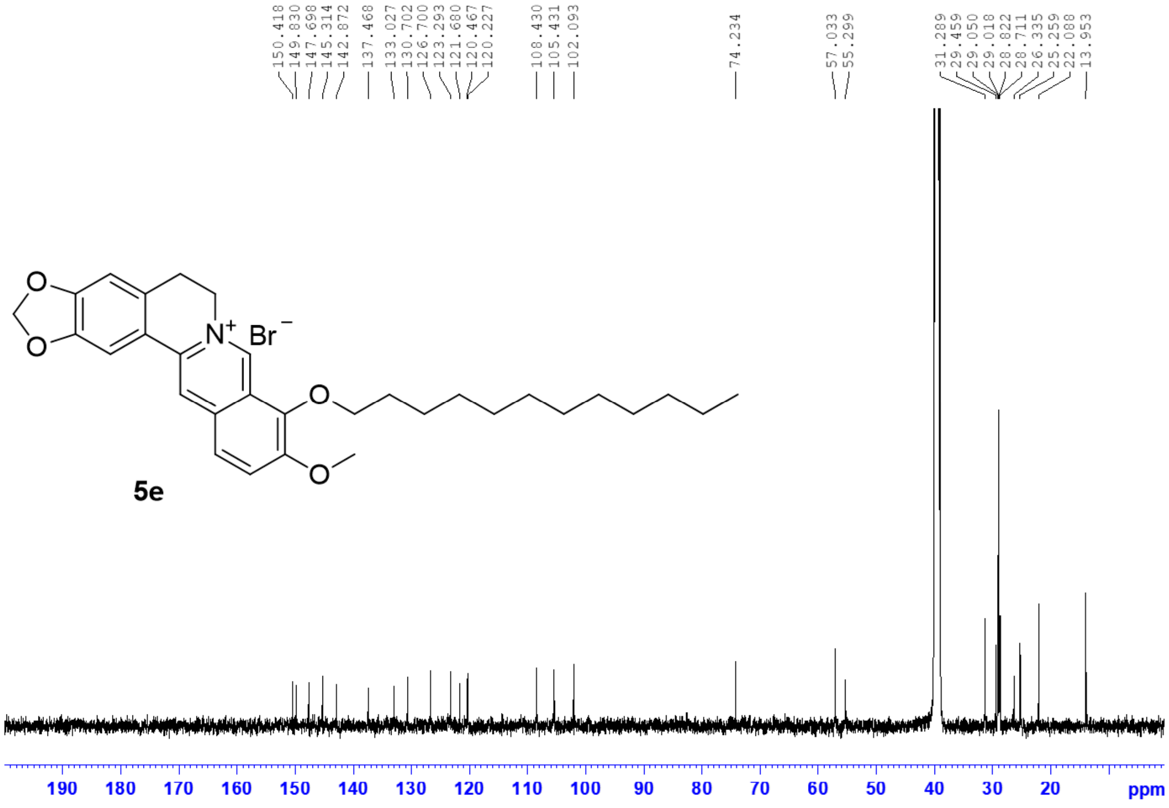

(**B**)

Figure S9. (A) ^{1}H NMR spectra of 9-*O*-dodecylberberine (**5e**); (B) ^{13}C NMR spectra of 9-*O*-dodecylberberine (**5e**).

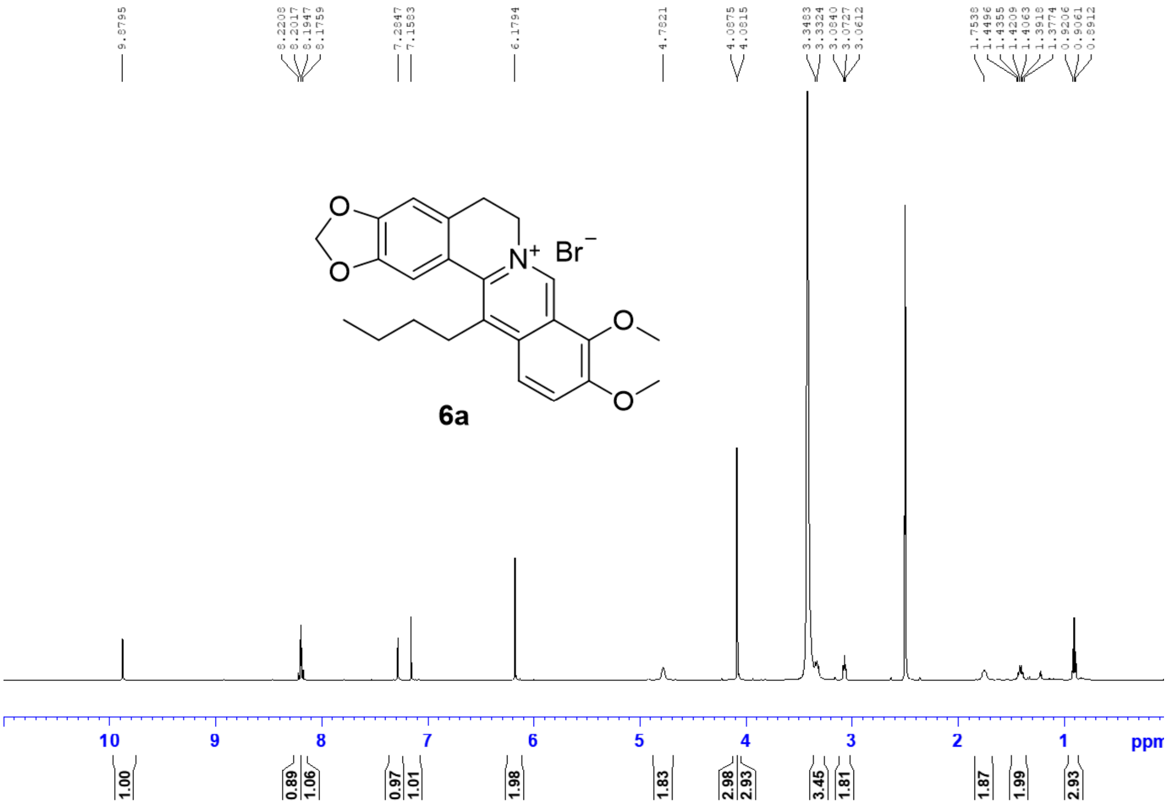

(**A**)

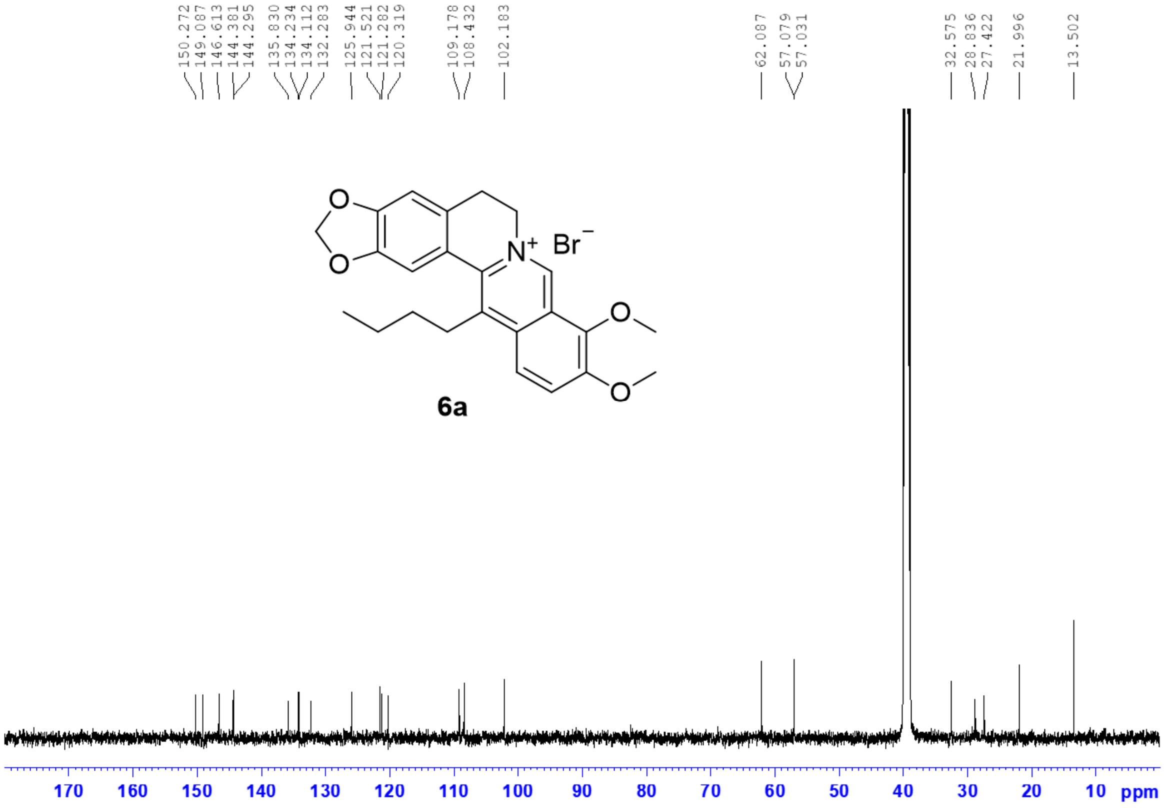

(**B**)

Figure S10. (A) ^{1}H NMR spectra of 13-butylberberine (**6a**); (B) ^{13}C NMR spectra of 13-butylberberine (**6a**).

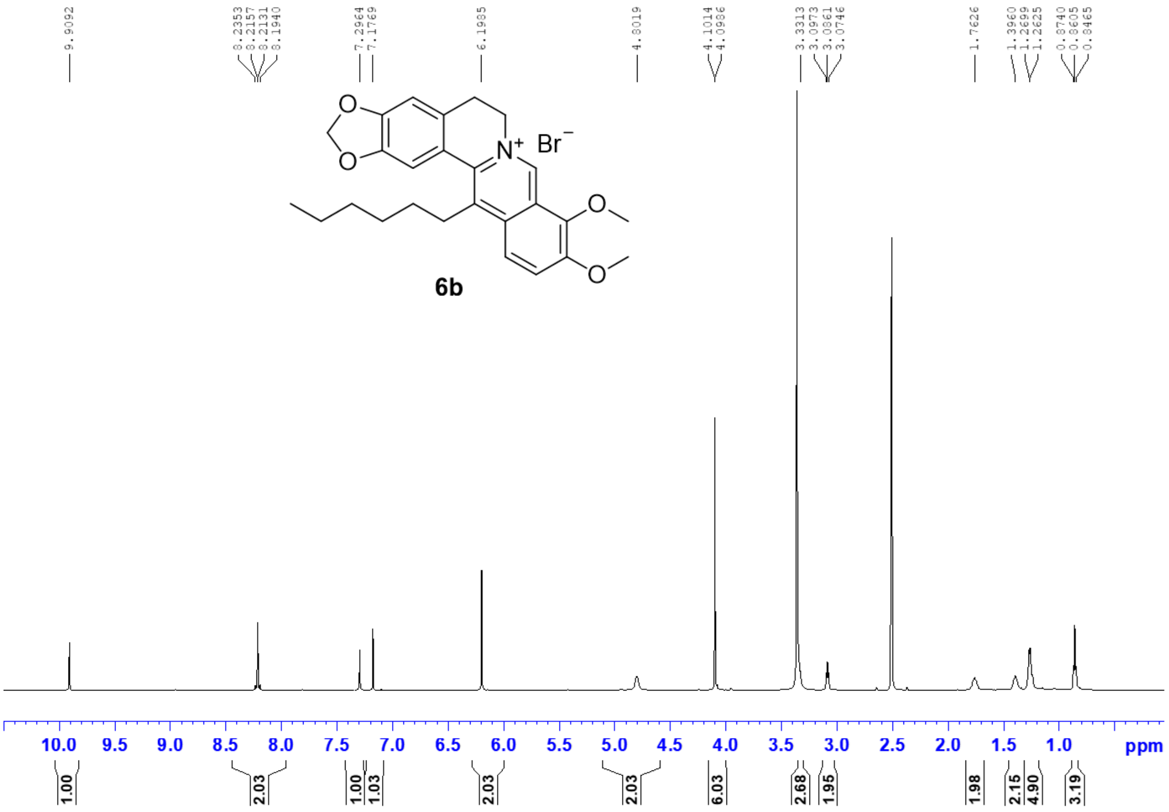

(**A**)

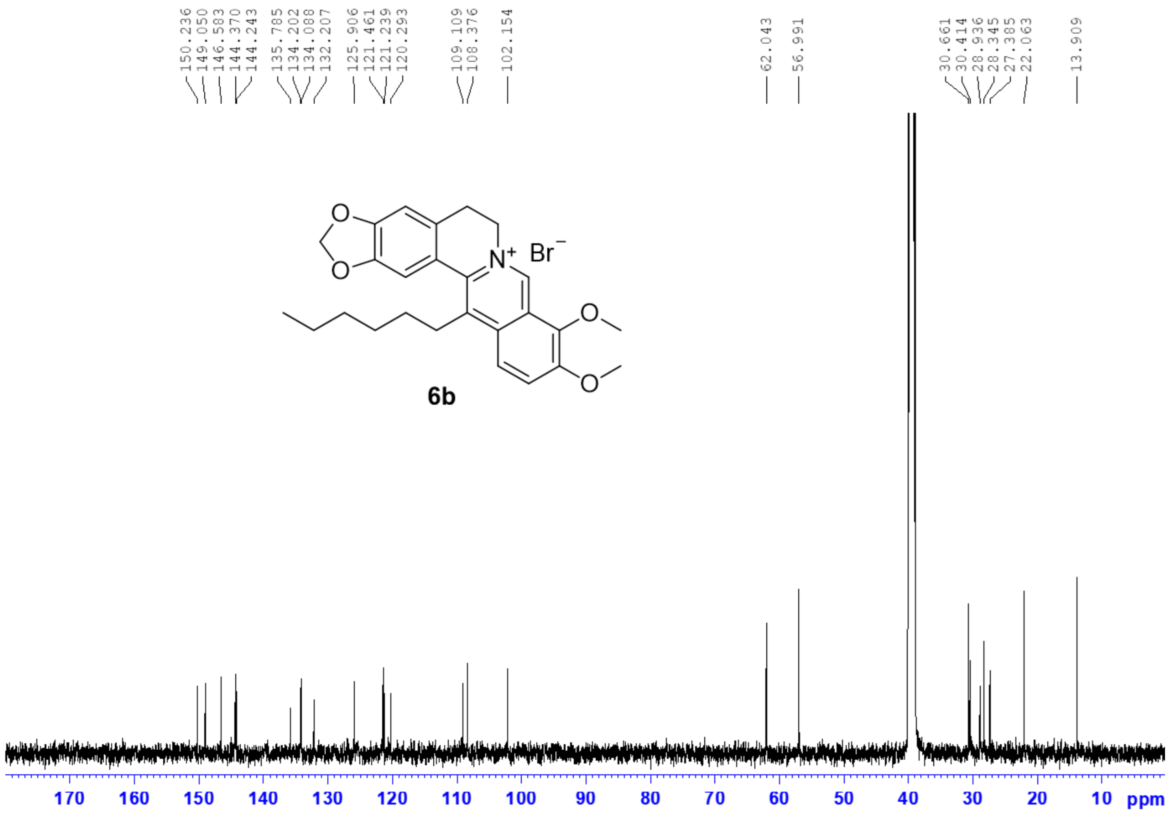

(**B**)

Figure S11. (A) ^{1}H NMR spectra of 13-hexylberberine (**6b**); (B) ^{13}C NMR spectra of 13-hexylberberine (**6b**).

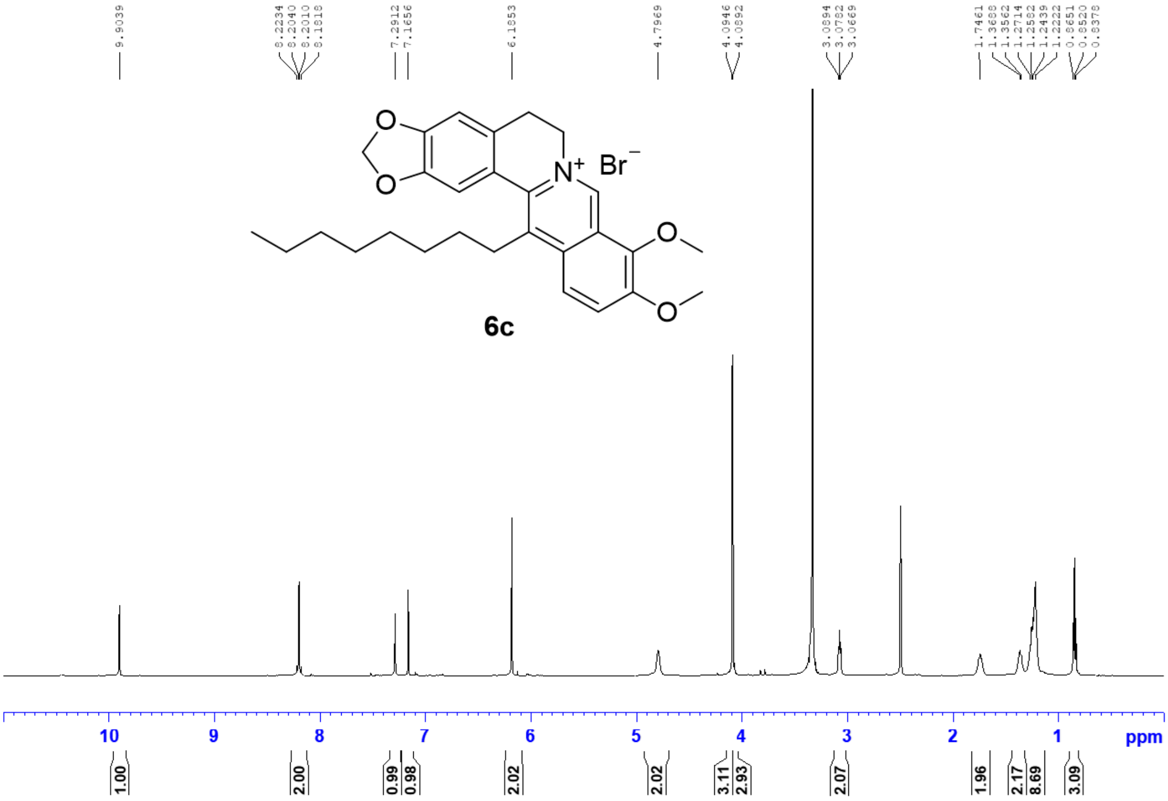

(**A**)

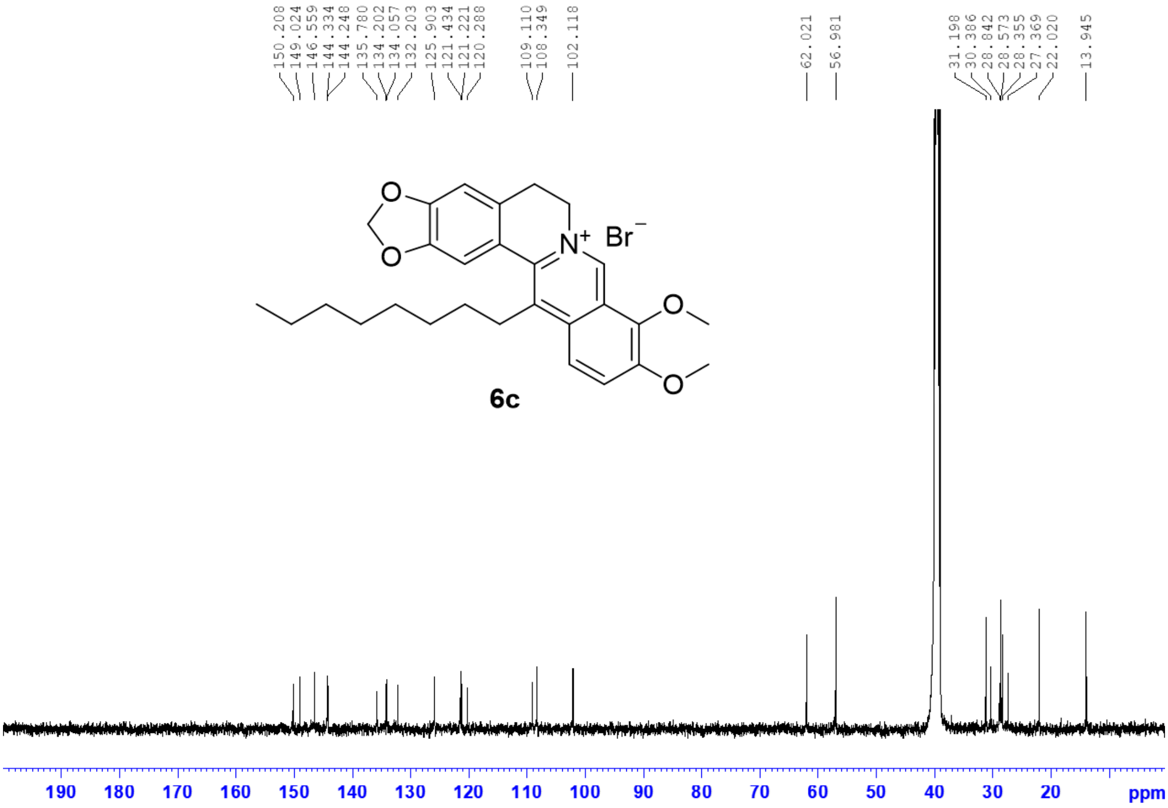

(**B**)

Figure S12. (A) ^{1}H NMR spectra of 13-octylberberine (**6c**); (B) ^{13}C NMR spectra of 13-octylberberine (**6c**).

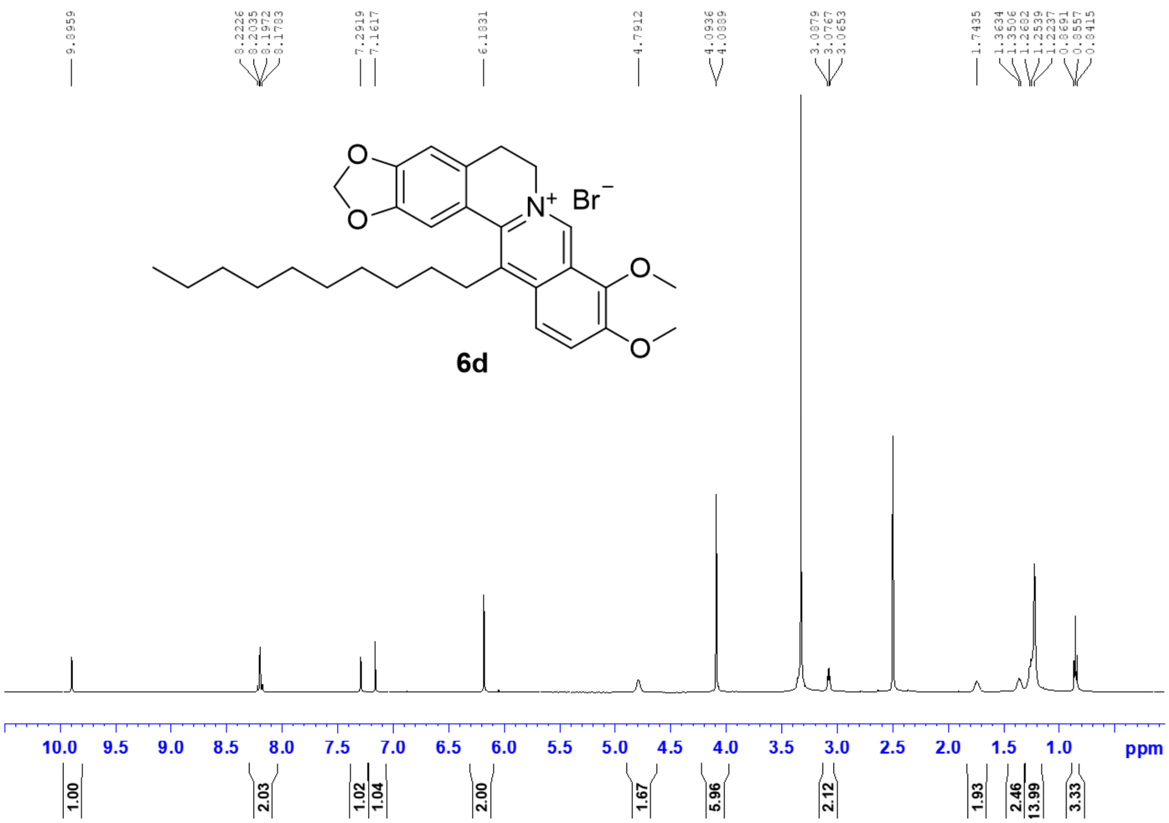

(**A**)

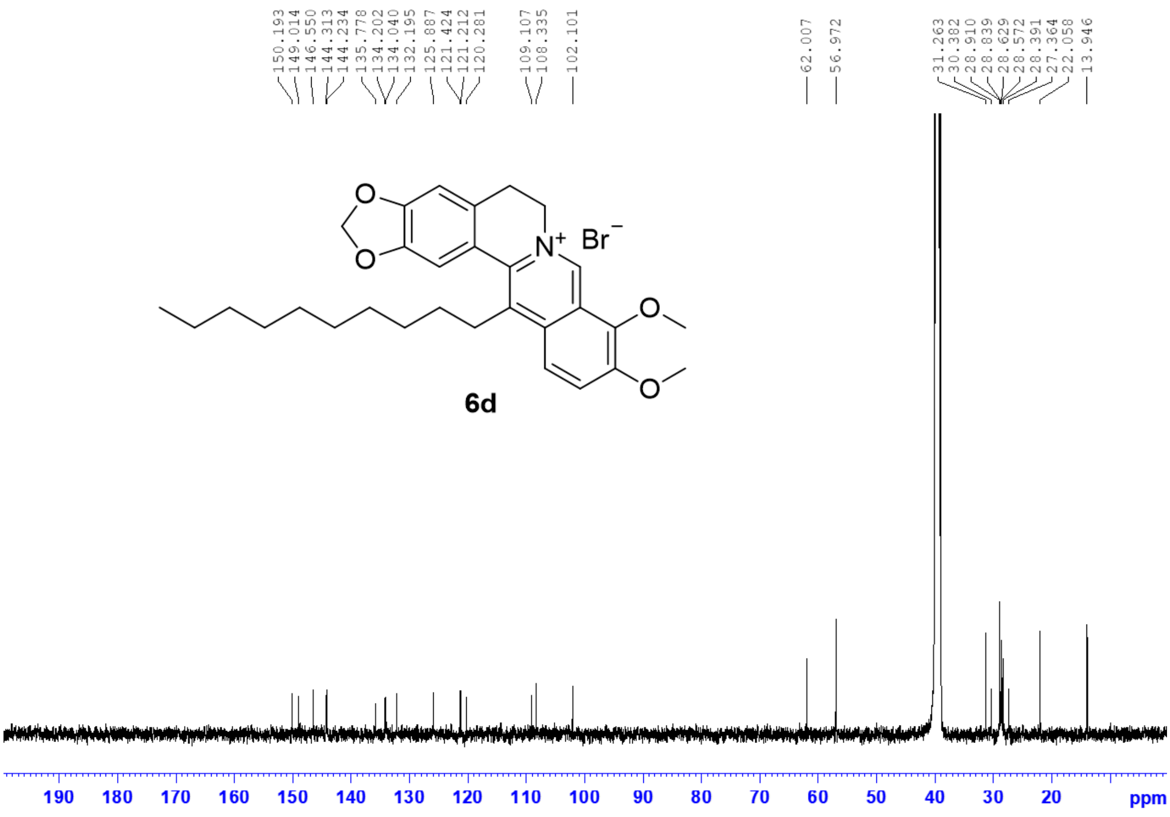

(**B**)

Figure S13. (**A**) ^{1}H NMR spectra of 13-decylberberine (**6d**); (**B**) ^{13}C NMR spectra of 13-decylberberine (**6d**).

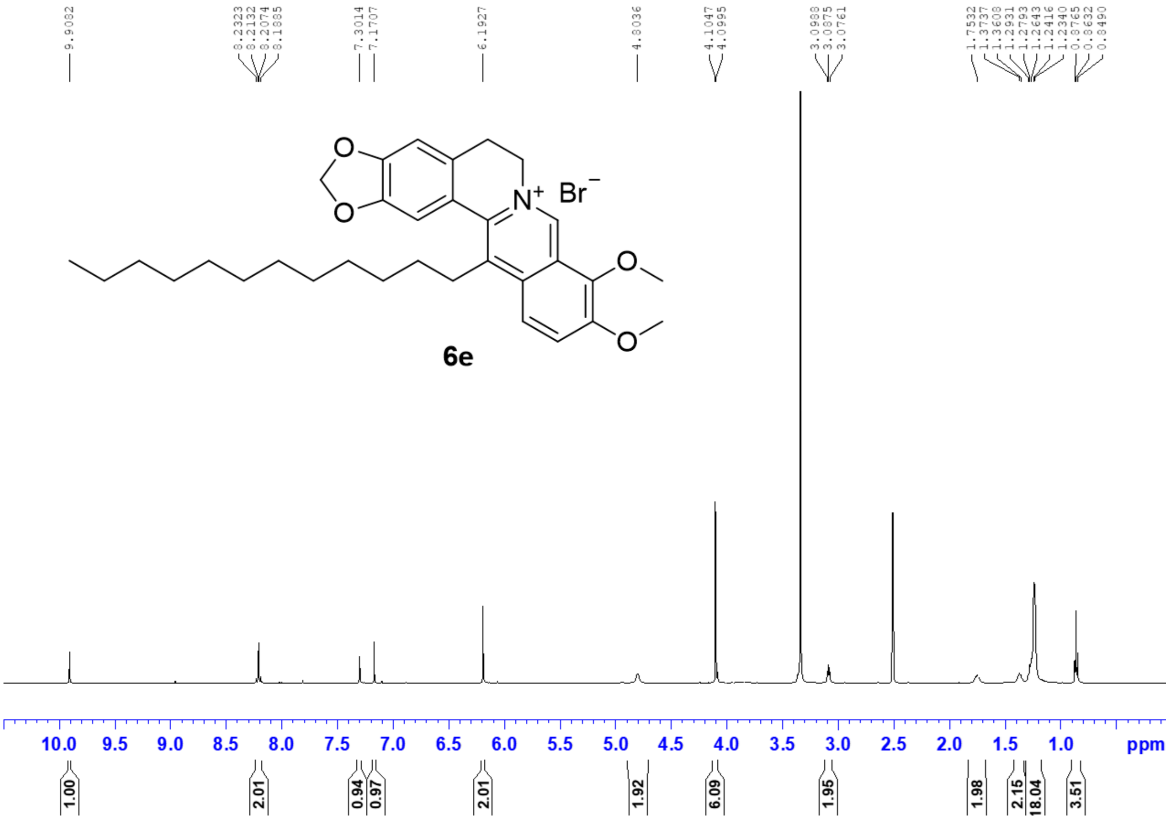

(**A**)

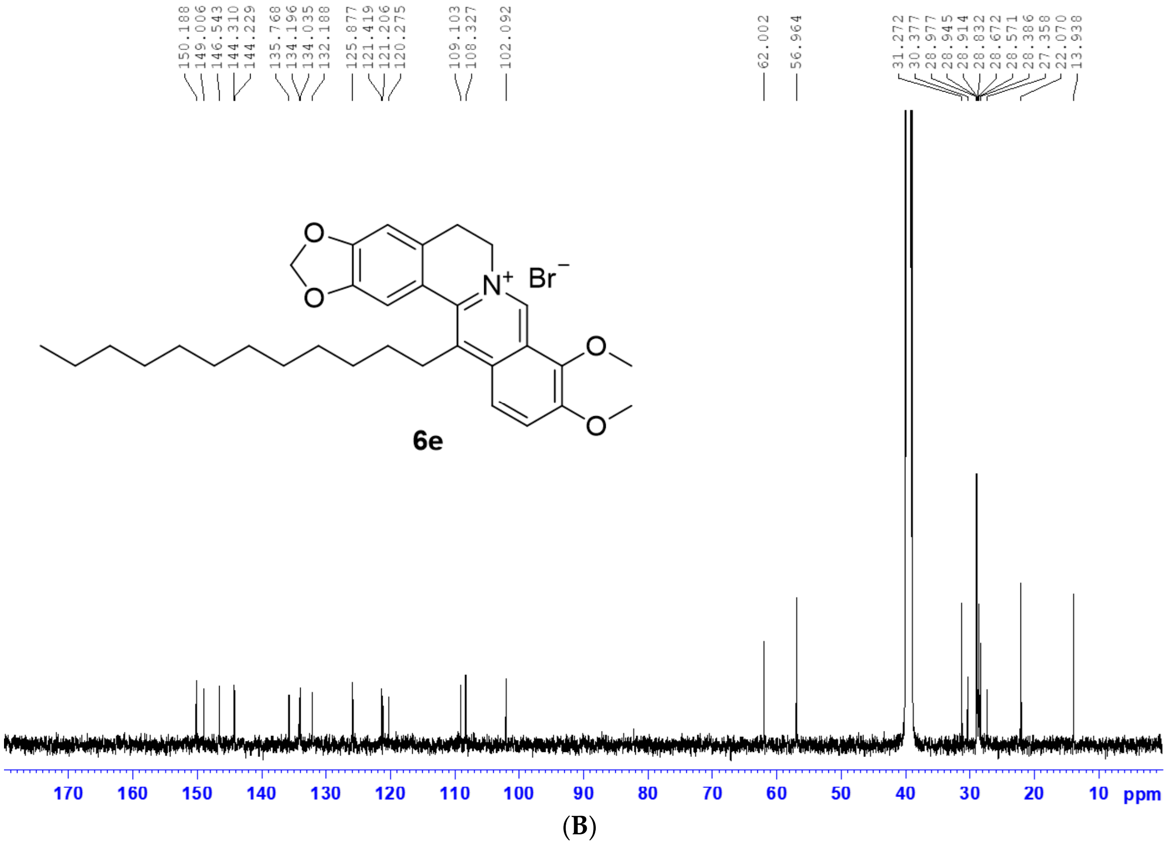

(**B**)

Figure S14. (A) ^{1}H NMR spectra of 13-dodecylberberine (**6e**); (B) ^{13}C NMR spectra of 13-dodecylberberine (**6e**).

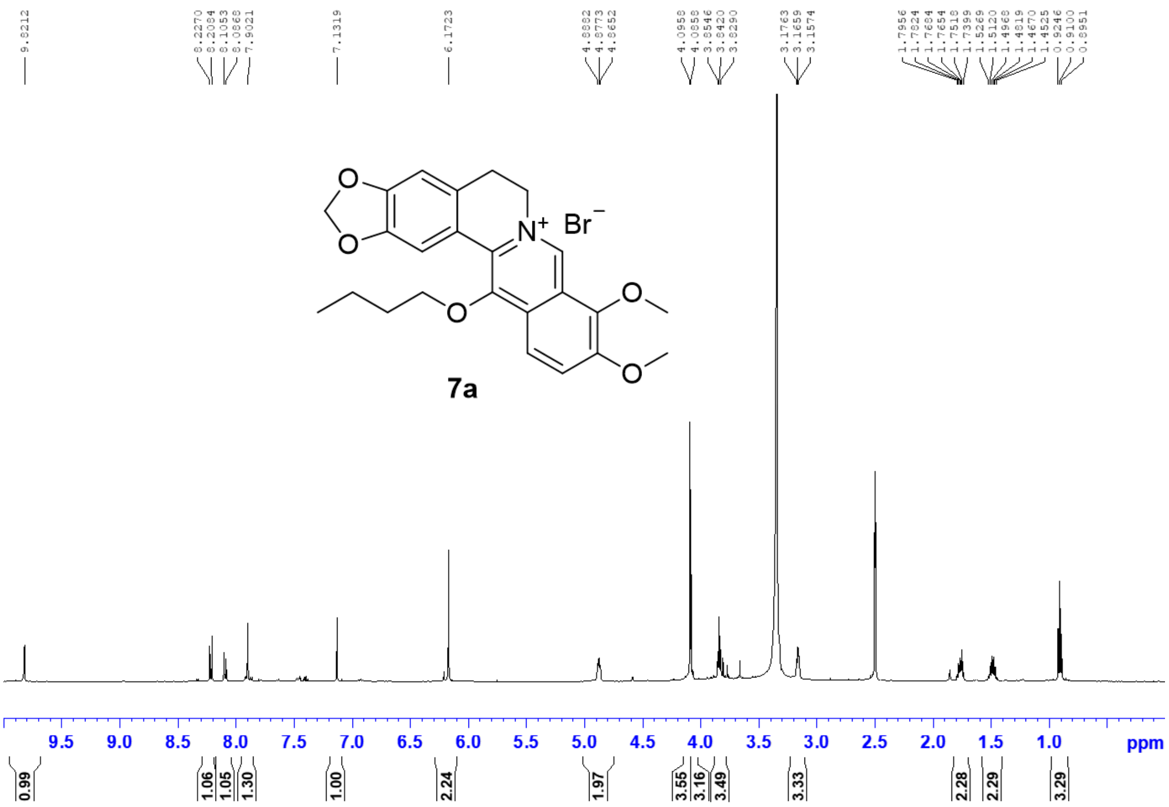

(**A**)

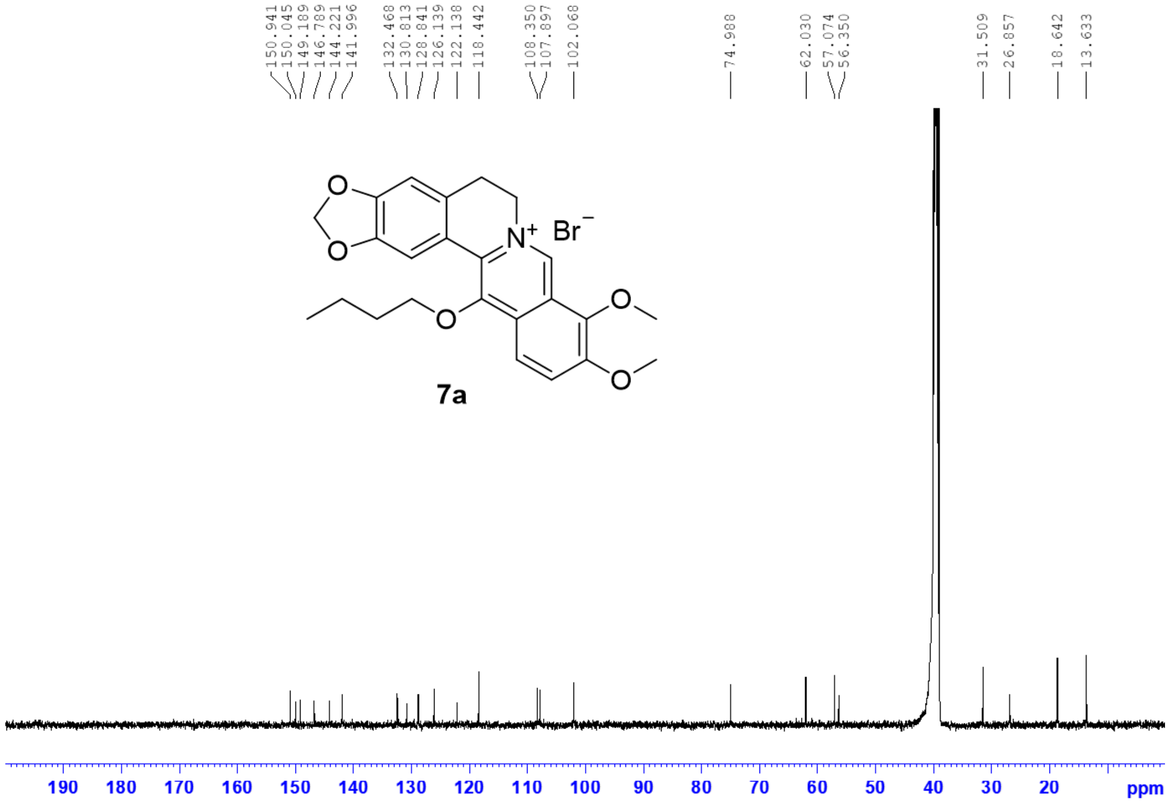

(**B**)

Figure S15. (A) ^{1}H NMR spectra of 13-*O*-butylberberine (**7a**); (B) ^{13}C NMR spectra of 13-*O*-butylberberine (**7a**).

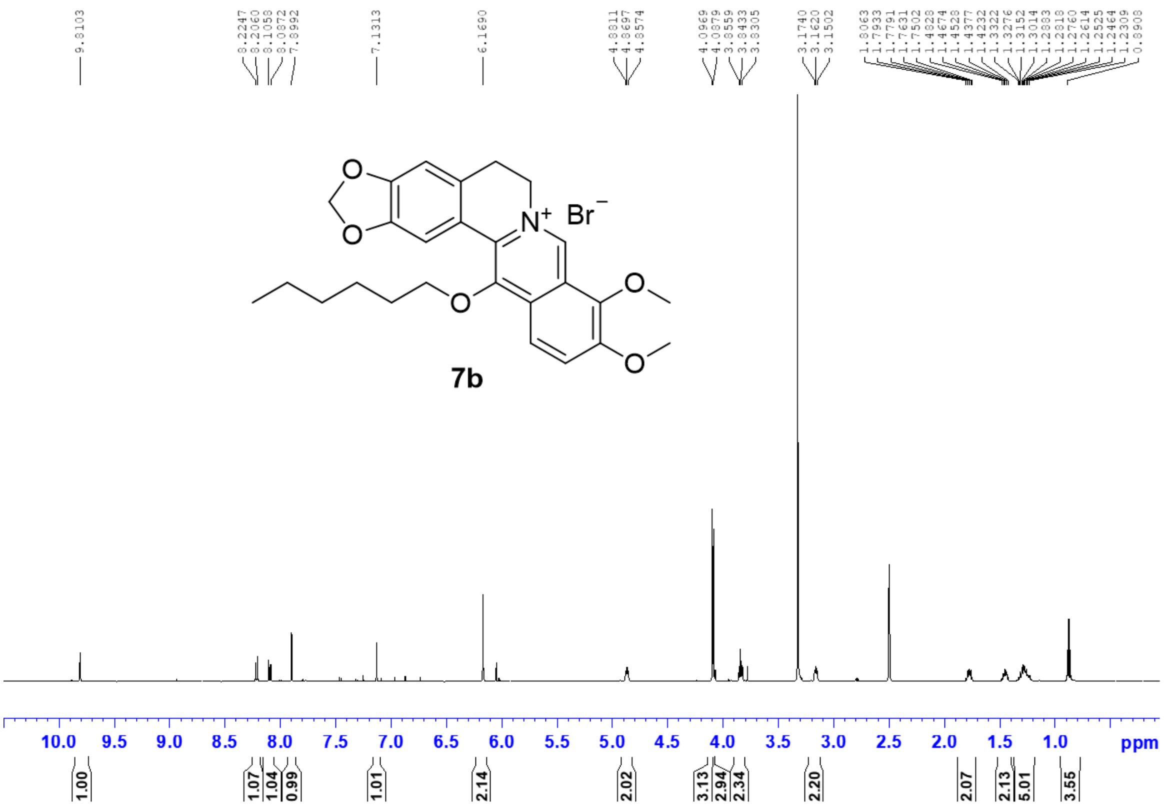

(**A**)

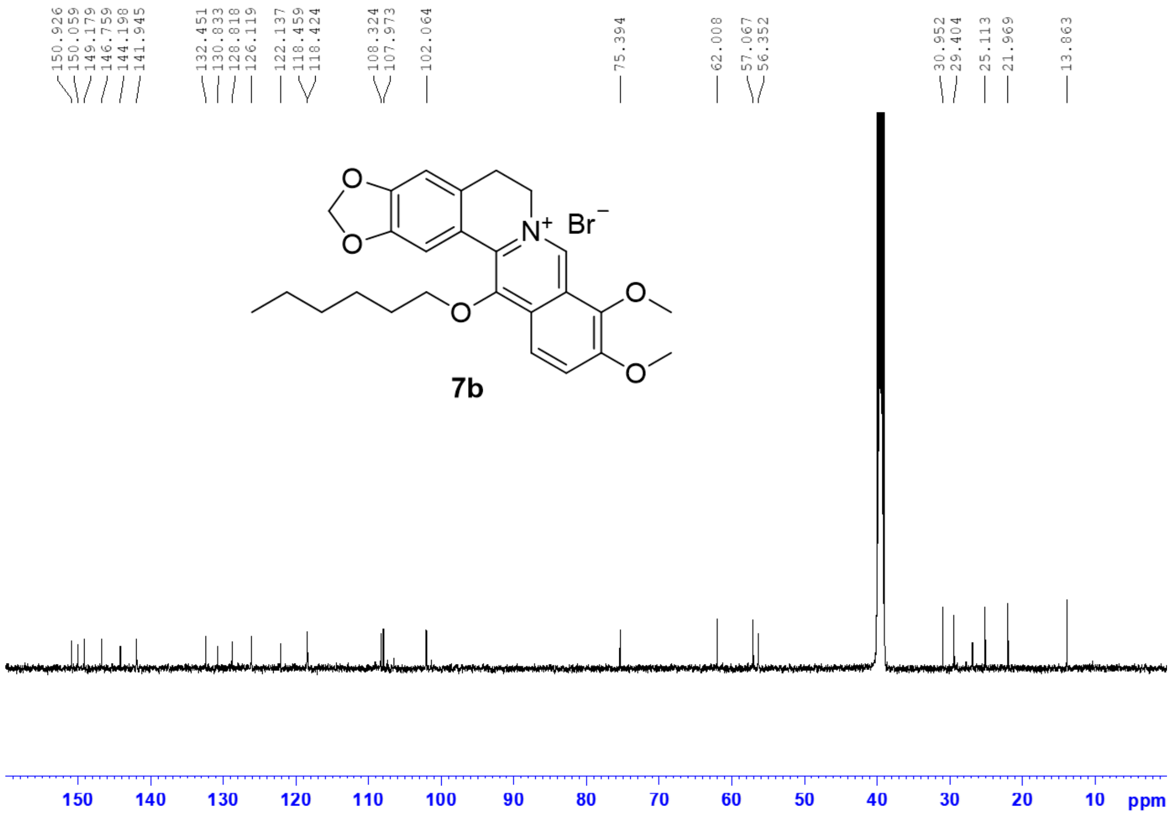

(**B**)

Figure S16. (A) ^{1}H NMR spectra of 13-*O*-hexylberberine (**7b**); (B) ^{13}C NMR spectra of 13-*O*-hexylberberine (**7b**).

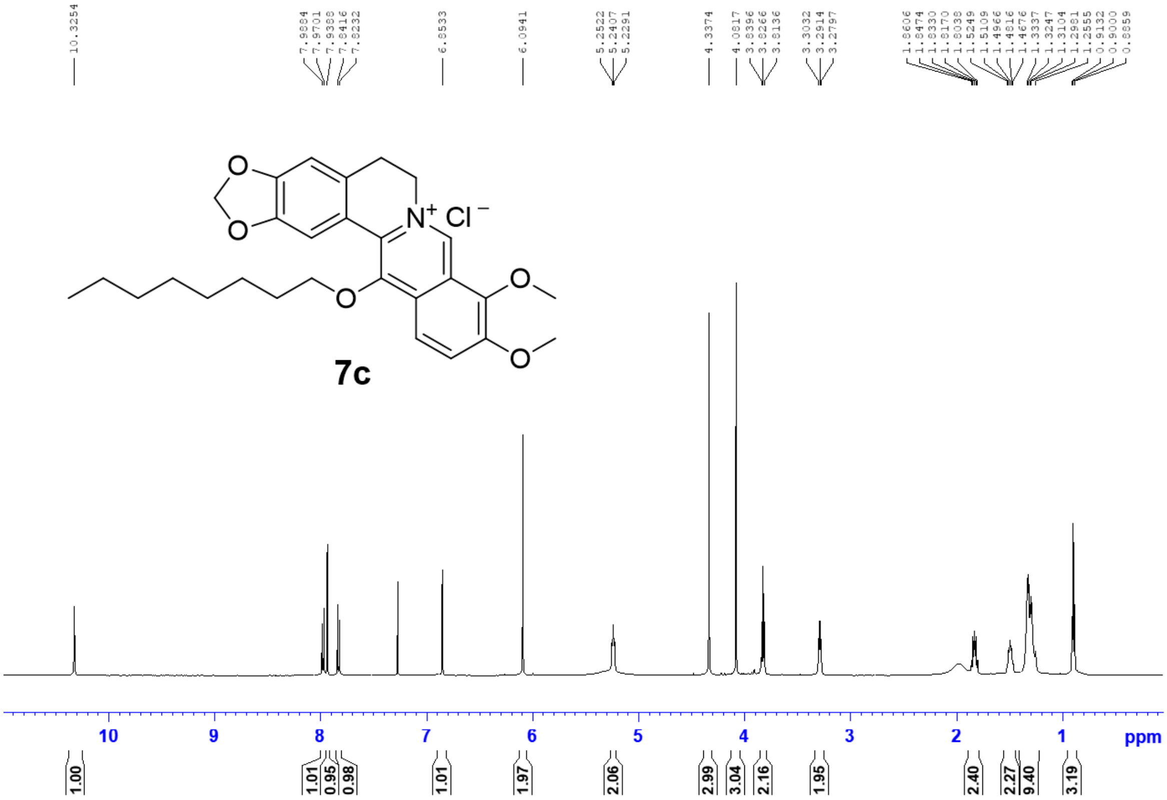

(**A**)

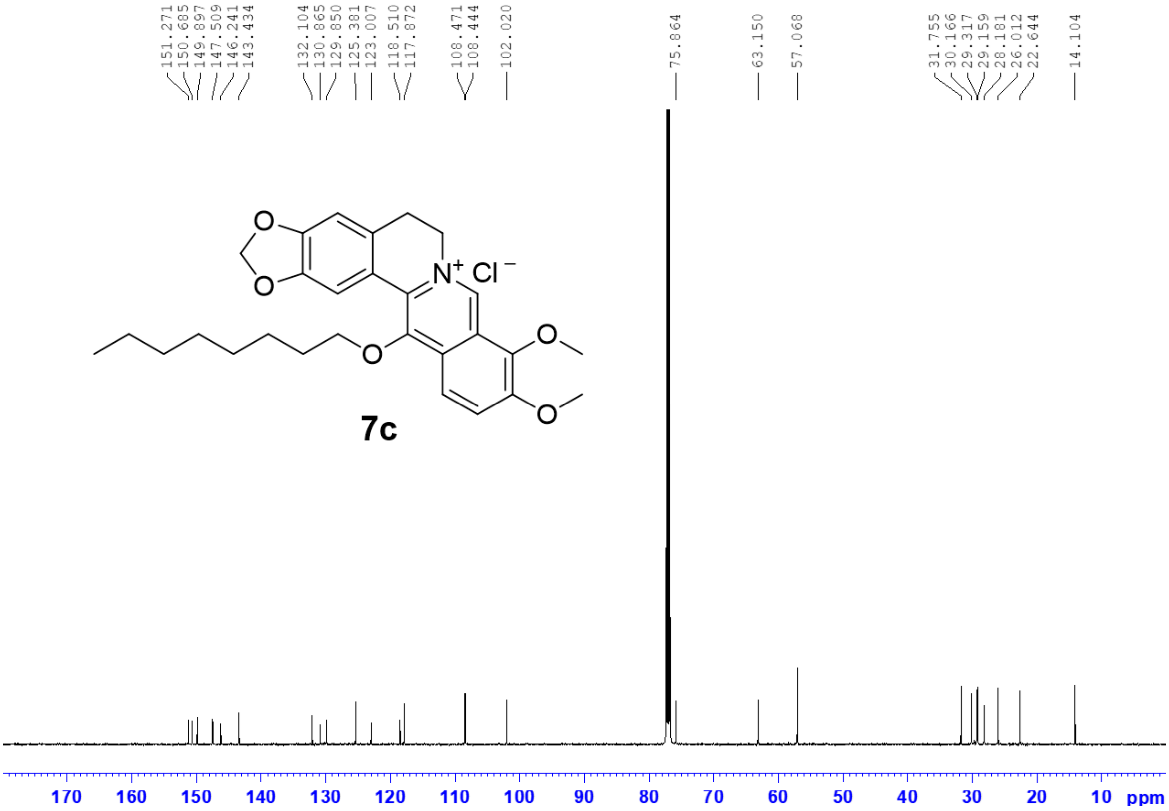

(**B**)

Figure S17. (A) ^{1}H NMR spectra of 13-*O*-octylberberine (**7c**); (B) ^{13}C NMR spectra of 13-*O*-octylberberine (**7c**).

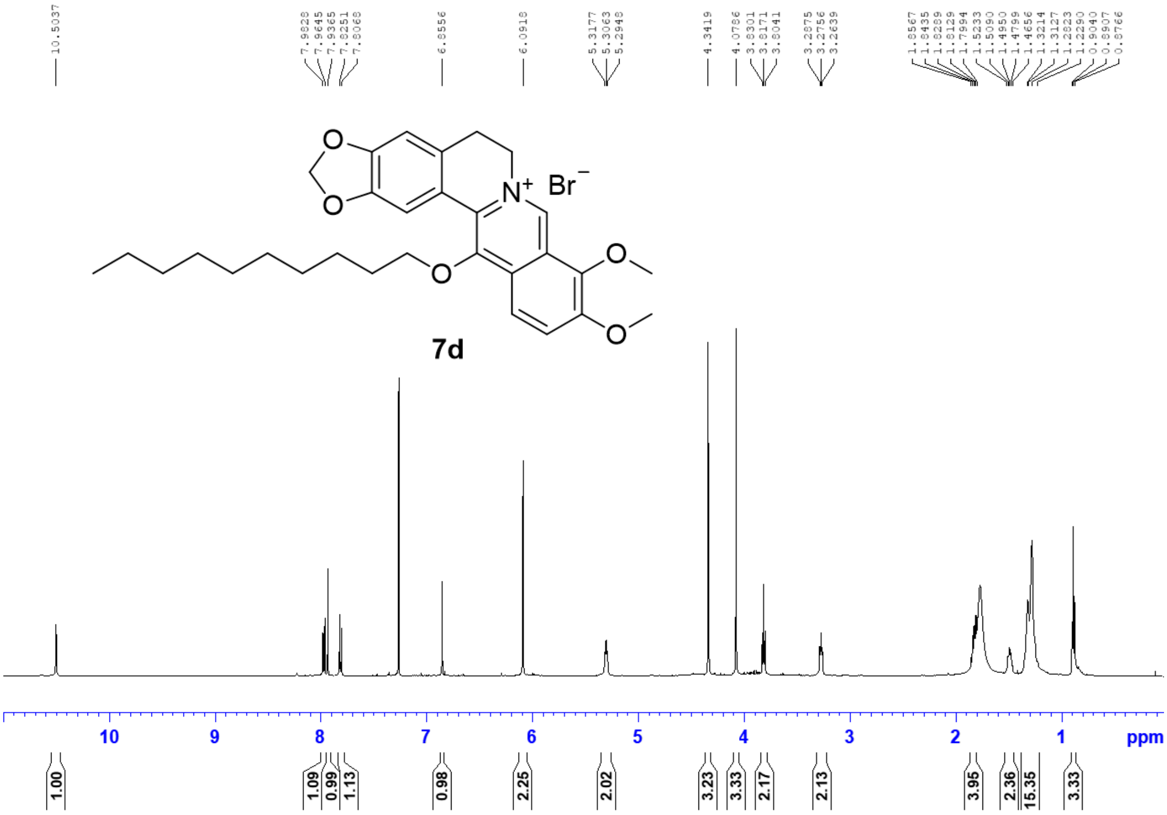

(**A**)

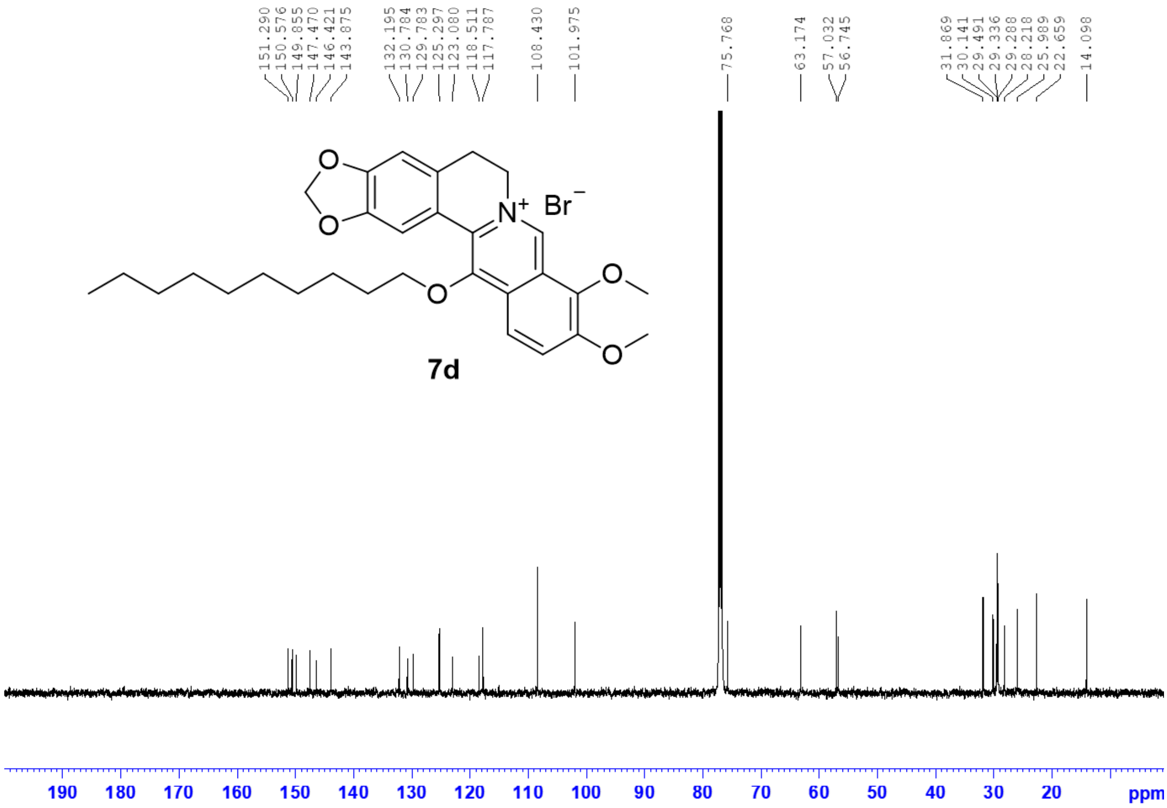

(**B**)

Figure S18. (A) ^{1}H NMR spectra of 13-*O*-decylberberine (**7d**); (B) ^{13}C NMR spectra of 13-*O*-decylberberine (**7d**).

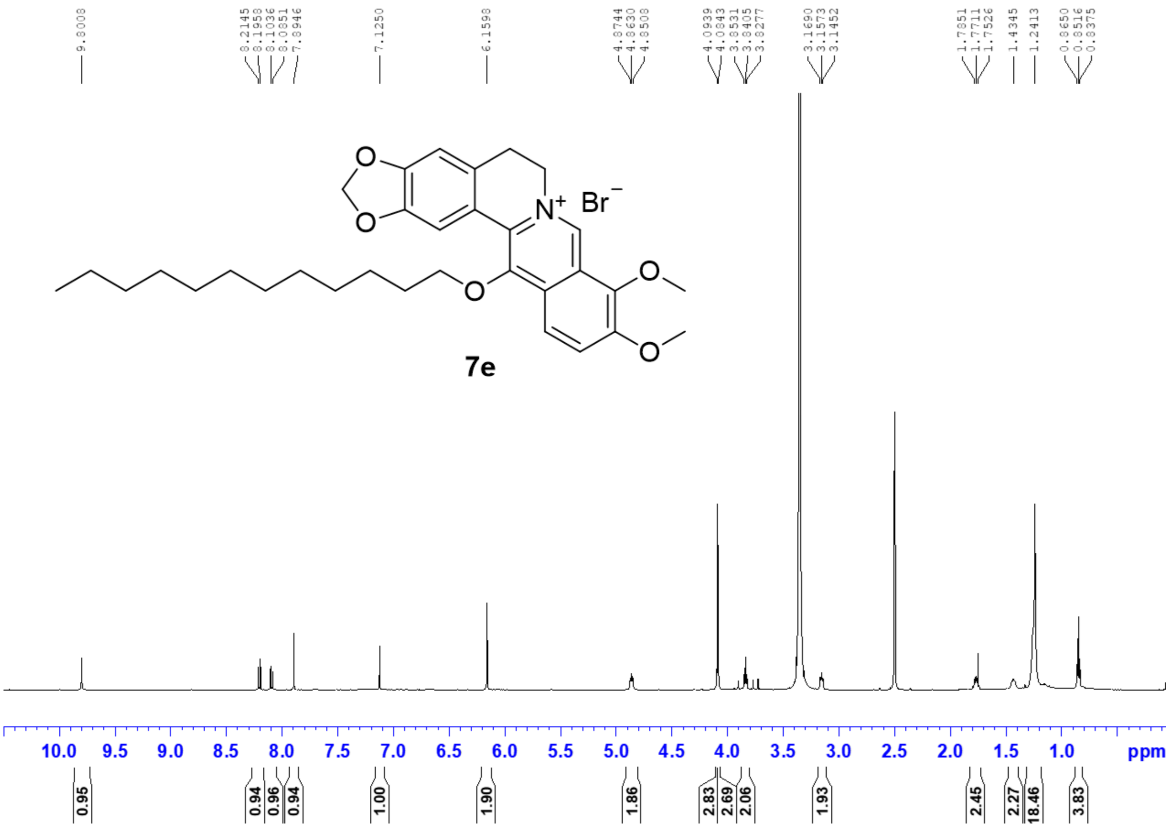

(**A**)

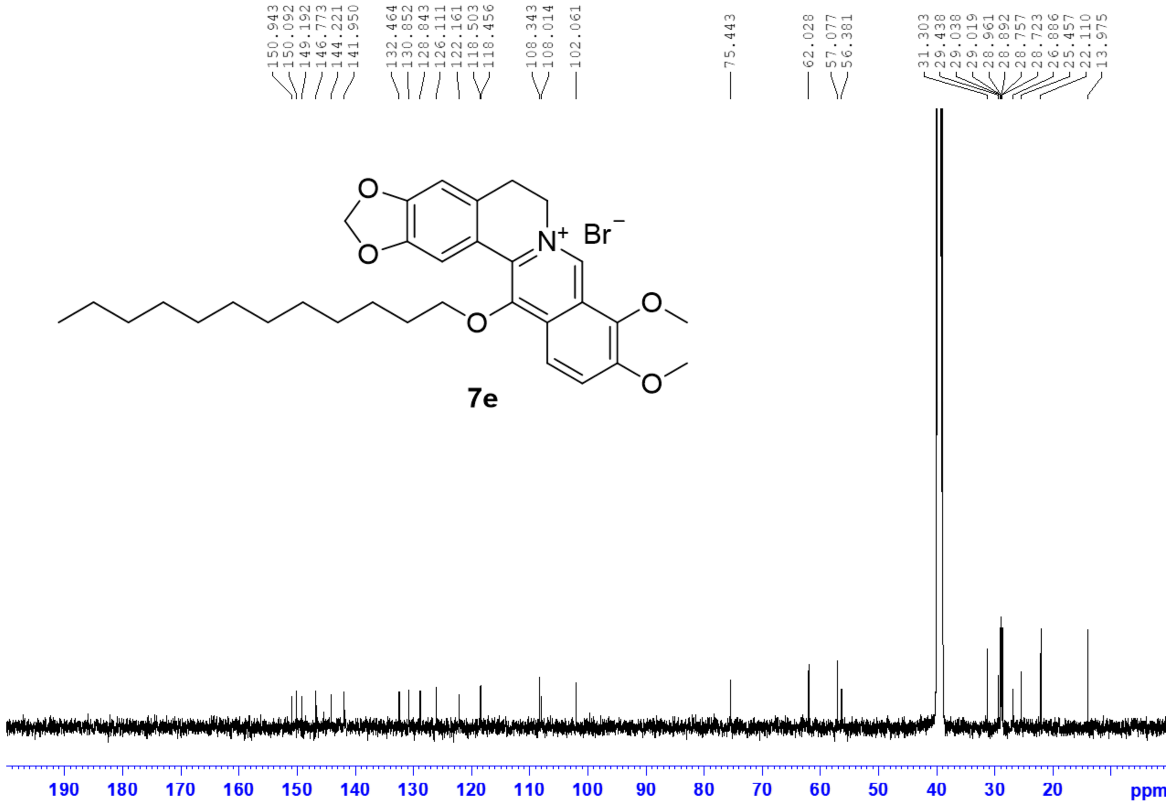

(**B**)

Figure S19. (A) ^{1}H NMR spectra of 13-*O*-dodecylberberine (**7e**); (B) ^{13}C NMR spectra of 13-*O*-dodecylberberine (**7e**).
